# Improving Lipid Profiles Through *Lactobacillus rhamnosus* Supplementation in Dyslipidemic Animal Models: A Systematic Review and Meta-Analysis

**DOI:** 10.3390/foods15030465

**Published:** 2026-01-29

**Authors:** Sungmin Chung, Jiill Jeong, Yeonwoo Park, Bogyeong Lee, Sumin Kang, Gwang-woong Go

**Affiliations:** Department of Food and Nutrition, Hanyang University, Seoul 04763, Republic of Korea; sungmin026103@gmail.com (S.C.); jiil.jeong119@gmail.com (J.J.); yeonwoo.park308@gmail.com (Y.P.); lbogyeong0313@gmail.com (B.L.); sumin.kang708@gmail.com (S.K.)

**Keywords:** dyslipidemia, *Lactobacillus rhamnosus*, *Lactobacillus casei*, *Lacticaseibacillus*, meta-analysis, systematic review

## Abstract

Dyslipidemia, characterized by elevated triglyceride (TG), total cholesterol (TC), and low-density lipoprotein cholesterol (LDL-C) levels, is a major cardiovascular risk factor. However, evidence regarding the lipid-modulating efficacy of *Lactobacillus rhamnosus* and *Lactobacillus casei* remains limited. This systematic review and meta-analysis (PROSPERO: CRD420251153531) evaluated their lipid-modulating effects in preclinical dyslipidemia models. A comprehensive search of four databases up to July 2025 identified 12 studies. Risk of bias was assessed using the SYRCLE tool. Random-effects meta-analyses were conducted to estimate standardized mean differences (SMDs) and 95% confidence intervals (CIs): probiotics significantly reduced TG (SMD: −1.38; 95% CI: from −1.92 to −0.84), TC (SMD: −0.85; 95% CI: from −1.20 to −0.42), and LDL-C levels (SMD: −1.59; 95% CI: from −2.16 to −1.02; all *p* < 0.001). In contrast, no significant effect was observed on high-density lipoprotein cholesterol levels (SMD: 0.18; 95% CI: from −0.35 to 0.72; *p* = 0.5044). Heterogeneity was moderate to substantial (I^2^ = 36–51%), although publication bias for TC and LDL-C suggests cautious interpretation of results. The lipid-lowering effects are likely mediated by bile salt hydrolase activity and short-chain fatty acid production along the gut–liver axis. These findings support *L. rhamnosus* as a potential adjunctive nutritional strategy for dyslipidemia management.

## 1. Introduction

Dyslipidemia has a high global prevalence and is recognized as a major risk factor for chronic diseases [1]. The condition is characterized by abnormal blood lipid profiles, including elevated triglyceride (TG), total cholesterol (TC), and low-density lipoprotein cholesterol (LDL-C) levels or decreased high-density lipoprotein cholesterol (HDL-C) levels [2]. Statins are effective in lowering LDL-C; however, they have limitations beyond myalgia, including contraindications and drug–drug interactions in some patients [3]. Residual cardiovascular risk can persist despite intensive statin treatment, and long-term adherence is often suboptimal, supporting the need for additional strategies [4,5]. Consequently, a growing interest exists in the use of safe nutritional strategies and nutraceuticals for the management of metabolic disorders [6]. Therefore, probiotic interventions have emerged as potential alternative therapeutic approaches [7]. Notably, within the lactic acid bacteria (LAB), the genus *Lacticaseibacillus* comprises species formerly classified as *Lactobacillus* before taxonomic reclassification in 2020 [8]. Gut microbiota plays a pivotal role in lipid metabolism, and its modulation is promising for managing dyslipidemia [9]. Recent studies demonstrate that probiotics can ameliorate dyslipidemia by modulating gut microbiota composition, altering bile acid metabolism, and enhancing cholesterol homeostasis [10,11]. The lipid-lowering effects of probiotics are largely attributed to their metabolic functions, particularly bile salt hydrolase (BSH) modulation and short-chain fatty acids (SCFAs) production, which enhance gut environment and restore microbial balance [12]. SCFAs, such as propionate and acetate, are transported to the liver via portal vein and suppress hepatic lipogenesis [13,14]. BSH produced by LAB induces the hydrolytic deconjugation of bile acids, reducing their intestinal reabsorption and enterohepatic recirculation while promoting fecal excretion [15]. This process increases hepatic cholesterol utilization for de novo bile acid synthesis to compensate for bile acid loss [16]. Moreover, bile acid deconjugation reduces cholesterol solubilization, thereby limiting intestinal cholesterol absorption [17]. Enhanced bile acid excretion reduces circulating cholesterol levels [18].

Within this genus, *Lacticaseibacillus rhamnosus* (*L. rhamnosus*) and *Lacticaseibacillus casei* (*L. casei*) are well characterized and extensively studied for their remarkable probiotic properties [19]. These strains were chosen based on their distinct yet synergistic mechanisms within the gut-liver axis [20,21]. Specifically, *L. rhamnosus* targets hepatic lipid metabolism via AMPK activation [22], whereas *L. casei* enhances intestinal integrity to reduce systemic endotoxemia [23]. This dual-focus approach supports a systematic review of these *Lacticaseibacillus* strains to elucidate their individual roles in managing dyslipidemia [24]. This study explored the potential of *L. rhamnosus* and *L. casei* to ameliorate dyslipidemia through regulation of the gut microbiota–bile acid–cholesterol axis, thereby maintaining lipid and cholesterol homeostasis. Although individual studies have reported these effects, a comprehensive synthesis remains lacking. Demonstrating the preclinical effectiveness of these strains is an essential step in confirming their potential for clinical translation in managing human dyslipidemia. This study hypothesized that supplementation with *L. rhamnosus* and *L. casei* can considerably improve dyslipidemia-related parameters, including blood TG, TC, LDL-C, and HDL-C. Accordingly, a systematic review and meta-analysis were conducted to evaluate the efficacy of these probiotic strains.

## 2. Materials and Methods

This systematic review and meta-analysis was conducted in accordance with the Preferred Reporting Items for Systematic Reviews and Meta-Analyses (PRISMA) guidelines [25]. The study protocol was prospectively registered in the International Prospective Register of Systematic Reviews (PROSPERO; registration number: CRD420251153531).

### 2.1. Search Strategy

A comprehensive literature search was performed in PubMed, the Cochrane Library, EMBASE, and Web of Science from database inception to 17 July 2025. The search strategy combined Medical Subject Headings and Embase Thesaurus terms with free-text keywords: (“gastrointestinal microbiome” OR “*Lactobacillus rhamnosus* (*L. rhamnosus*)” OR “*Lactobacillus casei* (*L. casei*)”) AND “dyslipidemias.”. Supplementary Table S1 provides detailed search strategies for each database. The search focused on *L. rhamnosus* and *L. casei* in accordance with the pre-registered PROSPERO protocol. The search was limited to peer-reviewed full-text articles, excluding grey literature due to insufficient extractable data for meta-analysis. Publication bias was assessed using funnel plots, Egger’s test, and trim-and-fill as a sensitivity analysis [26,27].

### 2.2. Criteria for Eligibility

Study eligibility was determined according to the Population, Intervention, Comparison, Outcome framework. The population included animal models with experimentally induced dyslipidemia. The intervention involved the administration of *L. rhamnosus* or *L. casei* as a single-strain probiotic. Single-strain was defined at the species level; therefore, combinations of different strains within *L. rhamnosus* or *L. casei* were classified as single-species interventions. The comparison comprised dyslipidemic control groups fed a high-fat diet without probiotic supplementation. Outcomes of interest included quantitative measurements of lipid-related profiles, specifically TG, TC, LDL-C, and HDL-C levels. The following exclusion criteria were applied: (1) studies in which *L. rhamnosus* and *L. casei* were administered in combination with other probiotic strains, (2) studies published only as abstracts or duplicate publications, (3) review articles, (4) studies not directly related to dyslipidemia, and (5) in vitro experimental studies. All relevant studies were imported into EndNote, and duplicates were removed using EndNote and Microsoft Excel. Two independent reviewers screened the titles, abstracts, and full texts using Microsoft Excel. Disagreements were resolved through consultation with a third reviewer [28].

### 2.3. Risk of Bias Assessment

The methodological quality of the individual trials was independently assessed by two reviewers using the Systematic Review Center for Laboratory Animal Experimentation (SYRCLE) risk-of-bias tool. The following domains were evaluated: sequence generation, allocation concealment, baseline characteristics, random housing, outcome assessor blinding, incomplete outcome data, selective reporting of outcomes, and other sources of bias. An additional domain assessing conflicts of interest and funding was included. Each domain was classified as having a low, high, or unclear risk of bias. The overall quality of each study was classified as low risk (all domains rated low), high risk (one or more domains rated high), or unclear (one or more domains rated unclear). Discrepancies between reviewers were resolved through discussion or, when necessary, third-party adjudication until a consensus was reached.

### 2.4. Data Synthesis and Analysis

All outcomes were treated as continuous variables and expressed as mean ± standard deviation (SD). When outcomes were reported as standard errors (SEs), SDs were calculated using the formula (SD = SE × √n) [29]. Effect sizes were calculated using Hedge’s g as the standardized mean difference (SMD) to correct for small sample bias [30]. This approach was necessary because lipid outcomes were presented in various measurement units, precluding unit conversion across all studies. To aid clinical interpretation, pooled SMDs were converted back to approximate mean differences in mg/dL. This was performed by multiplying the effect estimates and their 95% confidence intervals (CI) by a representative SD defined as the median control-group SD for each outcome [31]. Effect estimates were reported with corresponding 95% CI and 95% prediction intervals (PI). The 95% PI indicated the range of effects expected in a future comparable study and incorporated between-study heterogeneity [32]. For multi-arm studies with a single control group, the control group sample size was proportionally allocated across the intervention groups to avoid unit-of-analysis errors [33]. When multiple probiotic doses were reported within a single study, only the highest dose was selected for inclusion to minimize data duplication. This strategy preserved effect-size independence across the intervention arms. Following Cochrane guidelines, only one intervention arm per study was included to avoid double-counting shared controls and incorrectly narrowing the CI [33,34].

### 2.5. Meta-Analysis

Meta-analyses were conducted using R software (version 4.5.1; R Foundation for Statistical Computing, Vienna, Austria). Statistical significance was set at *p* < 0.05. Pooled effect estimates were calculated using a random-effects model to account for potential between-study heterogeneity [35]. Statistical heterogeneity across studies was assessed using the I^2^ statistic, with values greater than 50% indicating substantial heterogeneity [36]. Between-study variance (τ^2^) was estimated using the DerSimonian–Laird method, which is the default estimator implemented in the R meta package [37].

## 3. Results

### 3.1. Study Selection

**Figure 1:**
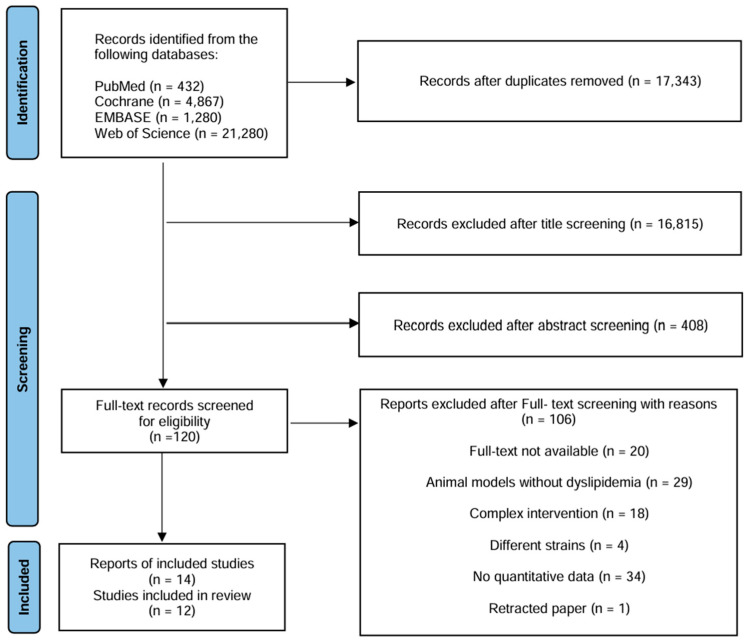
PRISMA flow diagram illustrating the study selection process for the systematic review and meta-analysis (update as of 15 January 2026).

A total of 27,859 records were initially identified through searches of online databases, including PubMed, Cochrane Library, EMBASE, and Web of Science. After importing the results into EndNote and Microsoft Excel, 17,343 studies were included. Title screening excluded 16,815 records that were deemed irrelevant to the study topic. Subsequent abstract screening led to the removal of 408 additional studies due to a lack of relevance to interventions or outcomes. During full-text assessment, 106 studies were excluded for the following reasons: full-text unavailable (*n* = 20), use of animal models not involving dyslipidemia (*n* = 29), intervention complexity (*n* = 18), use of different strains (*n* = 4), lack of quantitative data (*n* = 34), and retraction of the paper (*n* = 1). Initially, 14 studies met the inclusion criteria. However, upon detailed inspection, three studies by Arellano-García et al. [38] involved the same group of animals. To avoid data duplication, these studies were consolidated into a single study. Consequently, the final meta-analysis included 12 distinct studies (Figure 1).

### 3.2. Characteristics of the Included Studies

Table 1 summarizes the characteristics of the 12 included studies. Animal models comprised mice (*n* = 7) and rats (*n* = 5). Most studies used male animals, and only one study used female mice. Most animals were typically between 3 and 10 weeks of age, although one study did not report the age. Dyslipidemia and associated metabolic disorders were primarily induced using a high-fat diet (*n* = 8) or a high-fat/high-fructose diet (*n* = 2), while one study used a high-cholesterol diet, and another used a high-fat/high-cholesterol diet. Interventions primarily consisted of a single-strain *L. rhamnosus*, with one study incorporating *L. casei*. Probiotics were administered daily via oral gavage at doses ranging from 10^8^ to 10^10^ colony-forming units. Intervention durations varied between 4 and 24 weeks; three studies lasted <8 weeks, while nine studies extended to ≥8 weeks. Each study assessed at least one lipid parameter, with serum and plasma TG levels being the most frequently reported. TC and LDL-C levels were commonly measured, and several studies have additionally assessed HDL-C levels.

### 3.3. Risk of Bias in Included Studies

**Figure 2:**
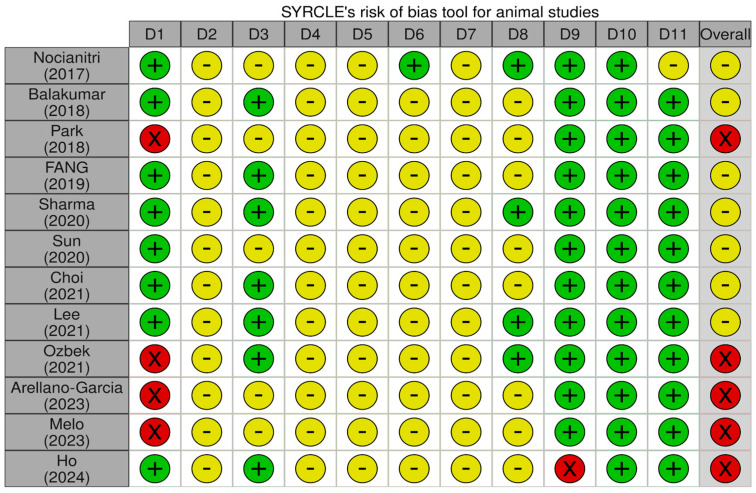
Risk of bias assessment in animal studies using the SYRCLE tool. Symbols indicate SYRCLE risk of bias judgments: “+” low risk, “−” unclear risk, and “X” high risk (green, yellow, and red, respectively) [22,38,39,40,41,42,43,44,45,46,47,48].

The SYRCLE risk of bias tool [49] was used to assess the methodological quality of the 12 studies (Figure 2). Overall, most studies were judged to have an unclear risk of bias across several domains, particularly due to insufficient methodological details provided in the original reports. Random sequence generation (Domain 1) was frequently inadequately described, with no clear information on how allocation sequences were generated or whether formal randomization procedures were applied. Domains 4–7 (random housing, blinding of caregivers/investigators, random outcome assessment, and blinding of outcome assessors) were predominantly rated as having an unclear risk of bias. These ratings largely reflect the inherent methodological limitations of animal experimental studies, in which full randomization and blinding, particularly during direct interventions, are often difficult or impracticable to implement.

### 3.4. Meta-Analysis of Lipid Outcomes

#### 3.4.1. Triglycerides

**Figure 3:**
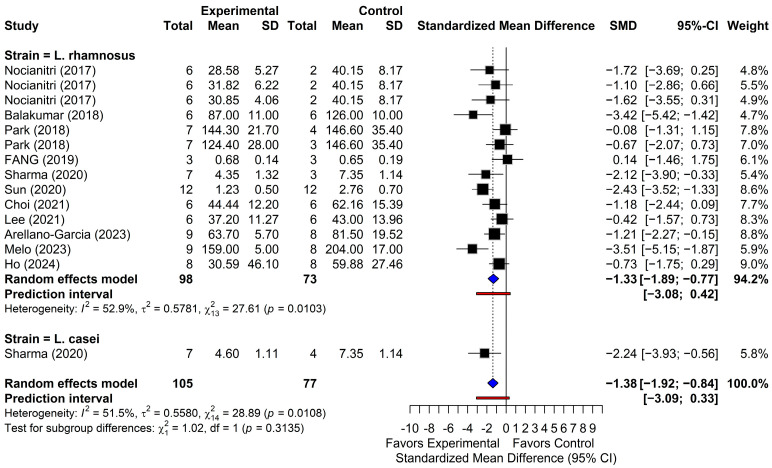
Forest plots of triglyceride (TG) outcomes. Black squares show individual study effect estimates (standardized mean difference, SMD) with 95% confidence intervals (CI), with square size proportional to study weight. Blue diamonds show pooled random-effects estimates. The vertical dotted line marks the null effect (SMD = 0), and the red line shows the prediction intervals (PI) [22,38,39,40,41,42,43,44,45,47,48].

Fifteen comparisons from 12 studies were evaluated and reported for TG levels. As shown in Figure 3, the random-effects model indicated that supplementation with *L. rhamnosus* and *L. casei* significantly reduced TG levels (SMD = −1.38; 95% CI, from −1.92 to −0.84; *p* < 0.001), with moderate to substantial heterogeneity across studies (I^2^ = 51.5%; *p* < 0.05). In original units, this pooled effect corresponds to an approximate TG reduction of 23.5 mg/dL (95% CI, from 14.4 to 32.6). The 95% PI (from −3.09 to 0.33) suggested a consistent TG-lowering effect across studies. Eight comparisons demonstrated statistically significant reductions in TG, with 95% CIs that did not cross the null value. Among the 15 comparisons, one study [41] reported a positive effect estimate (SMD = 0.14; 95% CI, from −1.46 to 1.75), whereas the remaining 14 comparisons demonstrated reductions in TG levels. Overall, the pooled analysis supported a robust decrease in TG levels following the *Lactobacillus* supplementation.

#### 3.4.2. Total Cholesterol

**Figure 4:**
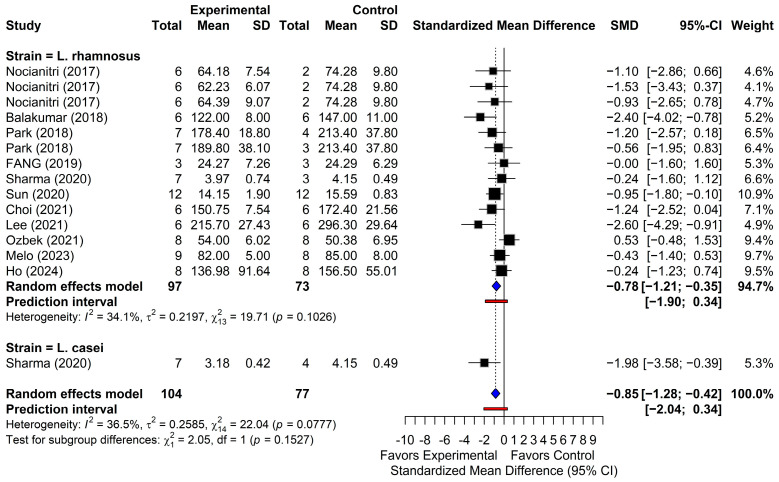
Forest plots of total cholesterol (TC) outcomes. Black squares show individual study effect estimates (standardized mean difference, SMD) with 95% confidence intervals (CI), with square size proportional to study weight. Blue diamonds show pooled random-effects estimates. The vertical dotted line marks the null effect (SMD = 0), and the red line shows the prediction intervals (PI) [22,39,40,41,42,43,44,45,46,47,48].

Fifteen comparisons were analyzed to assess the effects of probiotic supplementation on TC levels (Figure 4). The random-effects model indicated a significant reduction in TC following supplementation with *L. rhamnosus* and *L. casei* (SMD = −0.85; 95% CI, from −1.20 to −0.42; *p* < 0.001), with low to moderate heterogeneity among studies (I^2^ = 36.5%; *p* = 0.0777). When reconverted to the original units, this represented an estimated decrease in TC of 16.0 mg/dL (95% CI, from 7.8 to 24.1). The 95% PI (from −2.04 to 0.34) further supported a consistent downward trend in TC levels in subsequent investigations. Among the included comparisons, 14 reported negative SMDs, whereas one comparison (Ozbek, 2021) [46] showed a positive effect size (SMD = 0.53; 95% CI, from −0.48 to 1.53); however, its 95% CI crossed the null value, indicating no statistical significance. Collectively, these findings indicate a consistent TC-lowering effect of specific probiotic strains in animal models of dyslipidemia.

#### 3.4.3. Low-Density Lipoprotein Cholesterol

**Figure 5:**
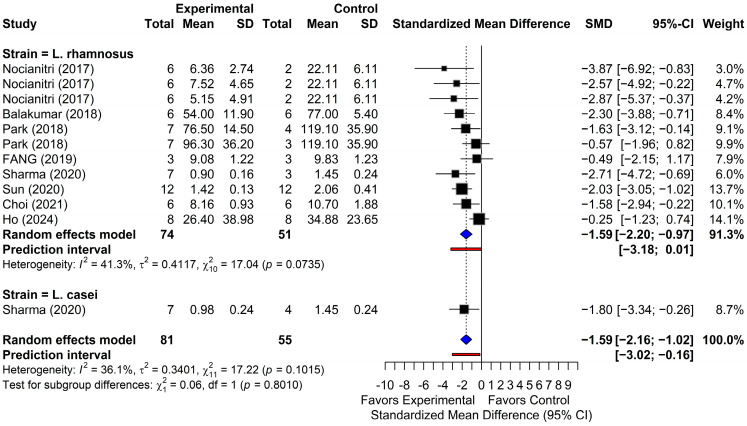
Forest plots of low-density lipoprotein (LDL-C) outcomes. Black squares show individual study effect estimates (standardized mean difference, SMD) with 95% confidence intervals (CI), with square size proportional to study weight. Blue diamonds show pooled random-effects estimates. The vertical dotted line marks the null effect (SMD = 0), and the red line shows the prediction intervals (PI) [22,39,40,41,42,43,44,48].

As shown in Figure 5, the pooled analysis of 12 comparisons revealed a significant reduction in LDL-C levels in the probiotic-treated groups compared with the controls. The random-effects model indicated a significant overall effect (SMD = −1.59; 95% CI, from −2.16 to −1.02; *p* < 0.001), with low to moderate heterogeneity among studies (I^2^ = 36.1%; *p* = 0.1015). In the clinical unit, this combined estimate indicated a decrease in LDL-C of 12.2 mg/dL (95% CI, from 7.9 to 16.6). The 95% PI (from −3.02 to −0.16) indicated that future studies are expected to show a reduction in LDL-C levels within this range. All comparisons reported negative SMDs, and nine demonstrated statistically significant reductions with 95% CIs that did not cross the null value. Overall, these results confirm the efficacy of probiotic interventions in significantly lowering LDL-C levels.

#### 3.4.4. High-Density Lipoprotein Cholesterol

**Figure 6:**
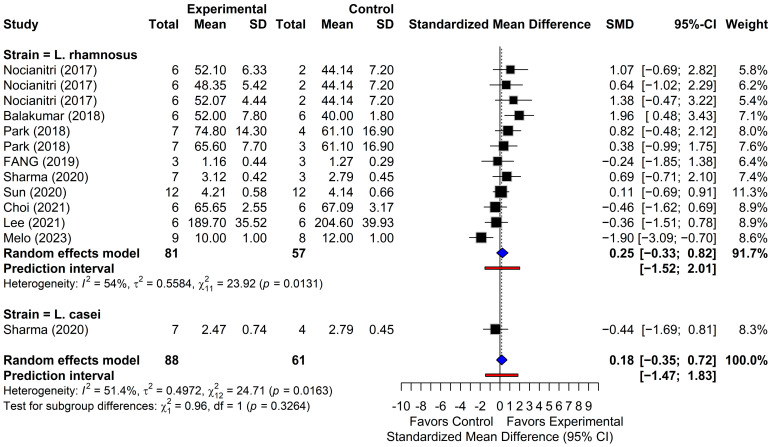
Forest plots of high-density lipoprotein (HDL-C) outcomes. Black squares show individual study effect estimates (standardized mean difference, SMD) with 95% confidence intervals (CI), with square size proportional to study weight. Blue diamonds show pooled random-effects estimates. The vertical dotted line marks the null effect (SMD = 0), and the red line shows the prediction intervals (PI) [22,39,40,41,42,43,44,45,47].

Thirteen comparisons from nine studies reported HDL-C outcomes (Figure 6). The random-effects model revealed a non-significant overall effect of probiotic supplementation on HDL-C levels (SMD = 0.18; 95% CI, from −0.35 to 0.72; *p* = 0.5044), with moderate to substantial heterogeneity among studies (I^2^ = 51.4%; *p* < 0.05). Converting the SMD back to its original units indicated an HDL-C increase of 1.3 mg/dL, which was not statistically significant (95% CI, from −2.5 to 5.2). The 95% PI (from −1.47 to 1.83) indicated that subsequent investigations are likely to show variable outcomes, ranging from decreased to increased HDL-C levels. Five comparisons reported effect sizes opposite to the pooled estimate. Except for two comparisons, all 95% CIs crossed the null value, indicating a lack of statistical significance. Balakumar et al. [40] reported a significant increase in HDL-C (SMD = 1.96; 95% CI: from 0.48 to 3.43), whereas Caroline de Oliveira Melo et al. [47] observed a significant decrease (SMD = −1.90; 95% CI, from −3.09 to −0.70). Overall, these findings did not support a consistent or statistically significant effect of probiotic supplementation on HDL-C levels.

#### 3.4.5. Subgroup Analyses by Intervention Duration, Animal Species, and Diet Type

To investigate potential variations among studies, subgroup analyses were performed for all primary outcomes based on intervention duration (<8 vs. ≥8 weeks), animal species (mice vs. rats), and diet type. The pooled estimates specific to each subgroup are detailed in Table 2, with the corresponding forest plots shown in Supplementary Figures S4–S6. As indicated in Table 2, significant differences between subgroups were found for TG by diet type (*p* < 0.05) and for LDL-C by animal species (*p* < 0.05). Conversely, no statistically significant differences were observed for TC or HDL-C across the moderators examined. Overall, these findings suggest that the lipid-modulating effects of these probiotics were largely consistent across various experimental conditions, with limited evidence of effect modification by diet composition for TG and by species for LDL-C.

### 3.5. Publication Bias Assessment

Potential publication bias was assessed using visual assessment of funnel plot symmetry and Egger’s regression test [50]. Supplementary Figure S1 shows the funnel plots of all four lipid parameters. The results of Egger’s test were consistent with those of the visual assessments, providing statistical verification of funnel plot symmetry. Significant publication bias was detected for TC (intercept = −2.567, *p* < 0.05) and LDL-C (intercept = −2.397, *p* < 0.05). In contrast, no evidence of publication bias was observed for TG (intercept = −2.337, *p* = 0.1561) and HDL-C (intercept = 2.439, *p* = 0.1725), as indicated by non-significant *p*-values.

## 4. Discussion

This meta-analysis demonstrates that *L. rhamnosus* supplementation improves lipid profiles in dyslipidemic animal models. Data for *L. casei* were limited because few eligible comparisons were available. Pooled effect sizes for TG, TC, and LDL-C confirmed significant reductions in lipid levels following probiotic intervention. The overall direction of effect across studies for these outcomes indicates consistency of the lipid-lowering effects across diverse experimental conditions. Moreover, the 95% PI for LDL-C suggests that future studies are likely to produce comparable beneficial effects, supporting the stability of these findings. Nonetheless, significant publication bias was detected for TC and LDL-C (Egger’s test, *p* < 0.05), which warrants cautious interpretation, as pooled estimates may overstate the actual effect sizes. Exclusion of grey literature may have contributed to the publication bias signals observed for TC and LDL-C. Trim-and-fill adjusted estimates are reported as sensitivity analyses, and the pooled effects warrant careful interpretation. Accordingly, further experimental investigations are needed to validate these results.

The consistent reduction in lipid parameters across independent studies strengthens the biological plausibility of probiotic supplementation as an adjunctive strategy for dyslipidemia management. The lipid-lowering outcomes observed in this meta-analysis can be explained by two principal pathways involving the gut–liver axis: bile acid-mediated cholesterol regulation and metabolite-driven suppression of lipogenesis [51]. Decreases in TC and LDL-C are consistent with bile acid-cholesterol regulation, including bile salt hydrolase activity of *Lacticaseibacillus* strains. TG reduction is consistent with microbiota-derived metabolites that suppress hepatic lipogenesis, whereas circulating HDL-C concentration is an insensitive readout of reverse cholesterol transport in rodent models. The BSH activity of *L. rhamnosus* promotes bile acid deconjugation [52], increasing fecal excretion of bile acids and stimulating hepatic conversion of cholesterol into new bile acids [53]. This pathway provides an explanation for the LDL-C reductions observed across studies, as hepatic cholesterol depletion upregulates LDL-C receptor expression and enhances LDL-C clearance [54]. In addition, evidence highlights low-density lipoprotein receptor-related protein 6 as a key regulator of LDL removal and potential therapeutic target modulated by probiotics [55]. Beyond BSH activity and SCFA production, preclinical studies have identified unique strain-specific molecular pathways underlying the lipid-modulating effects of these probiotics. *L. rhamnosus* exhibits anti-atherogenic properties by activating the nuclear receptor liver X receptor α in macrophages in vitro [56,57]. This activation upregulates cholesterol efflux transporters ABCA1 and ABCG1, thereby limiting foam cell formation and indirectly lowering circulating LDL-C levels [58,59]. Second, reductions in TG levels are mechanistically linked to SCFA production resulting from probiotic-induced gut microbiota modulation. Propionate and acetate inhibit hepatic lipogenesis through activation of the AMP-activated protein kinase pathway [60]. Given that inhibiting the mammalian target of rapamycin pathway suppresses de novo lipogenesis [61], probiotics may exert complementary metabolic effects through these signaling cascades. Furthermore, studies using *L. rhamnosus GG* supernatant have reported enhanced intestinal glucagon-like peptide secretion [62], which subsequently downregulates hepatic lipogenic genes, including *SREBP-1c* and *FAS* [63]. Although a direct in vivo causal relationship requires further validation, this mechanism represents a biologically plausible explanation consistent with TG reductions observed in the pooled analysis. Regarding *L. casei*, the limited data available in this meta-analysis preclude definitive conclusions. However, previous studies have proposed potential mechanisms involving hepatic proprotein convertase subtilisin/kexin type 9 regulation and toll-like receptor 4 signaling inhibition. Further experimental investigations are required to confirm these specific pathways and clarify the lipid-modulating effects of *L. casei* [54,64].

Regarding statistical robustness and variability, a significant overall reduction in TG levels was observed, with substantial heterogeneity (I^2^ = 51.5%), comparable to the heterogeneity observed for HDL-C (I^2^ = 51.4%). To explore potential sources of this heterogeneity, we conducted meta-regression analyses examining intervention duration and probiotic dose (log10 CFU) as moderators (Supplementary Table S2) [65]. For TG, intervention duration was not significantly associated with effect size (β = −0.03, *p* = 0.64), indicating that the TG-lowering effect of *L. rhamnosus* was consistent across different intervention durations within the studied range. To evaluate the influence of individual studies, Sensitivity analyses, including leave-one-out analysis (Supplementary Figure S2) and Baujat plot (Supplementary Figure S3) [66], confirmed that the overall direction and significance of the TG reduction remained stable even after excluding influential studies. Besides meta-regression, subgroup analyses (Table 2; Supplementary Figures S4–S6) showed consistent effect directions across outcomes, with effect sizes differing by diet type for TG and by species for LDL-C (*p* < 0.05). Several subgroups included few comparisons, so these contrasts should be interpreted with caution and do not support identifying a single most predictive model.

In contrast, the absence of a significant pooled effect on HDL-C does not indicate a lack of biological activity. Rather, moderate to substantial heterogeneity (I^2^ = 51.4%) suggests that inter-study variability may have attenuated detectable effects. Subgroup analysis by intervention duration (<8 vs. ≥8 weeks) yielded comparable effect sizes (Supplementary Figure S4); however, heterogeneity was substantially lower in the long-term subgroup (I^2^ = 23.2%) than in the short-term subgroup (I^2^ = 77.8%). Consistent with this observation, meta-regression analysis revealed that intervention duration accounted for approximately 14% between-study variance (Supplementary Table S2), although this association was not significant (β = 0.064, *p* = 0.24). Given the limited number of studies (k = 13) and modest HDL changes typically associated with probiotic interventions, the meta-regression may have been underpowered to detect significant moderator effects [67]. Accordingly, these findings should be interpreted as exploratory. Sensitivity analysis demonstrated the robustness of the HDL-C effect, as sequential exclusion of individual studies did not alter the overall effect direction (Supplementary Figure S2). Balakumar et al. [40] and Caroline de Oliveira Melo et al. [47] were major contributors to heterogeneity, as reflected in leave-one-out analysis and the Baujat plot (Supplementary Figure S3) [66]. Excluding these studies did not significantly affect the aggregated effects, suggesting that the overall conclusions were not dependent on any single influential study. Nevertheless, the lack of a significant HDL-C increase (SMD = 0.18, *p* = 0.5044) suggests that Circulating HDL-C is a static measure and might not effectively reflect changes in reverse cholesterol transport in rodent studies [68]. Consequently, cholesterol flux could be enhanced through ABCA1/ABCG1-mediated efflux without an increase in HDL-C levels, especially when liver uptake via SR-BI is elevated [69,70]. The physiology of rodent lipoproteins, which includes the absence of cholesteryl ester transfer protein, might further limit alterations in the circulating HDL-C pool [71,72]. Therefore, functional assessments like cholesterol efflux capacity could provide more valuable insights than merely measuring HDL-C levels [73]. Biologically, HDL-C metabolism is primarily controlled by host genetic factors and systemic metabolic regulation, rather than being predominantly modulated by the gut microbiota. Consequently, probiotics may have limited capacity to alter HDL-C compared with their effects on TG or LDL-C [74]. The modest HDL-C response may reflect insufficient intervention duration, particularly in animal studies [75]. Additionally, baseline HDL-C levels, genetic variability, and disease severity may influence responsiveness [76]. Collectively, these findings suggest meaningful HDL-C modulation through probiotic interventions may require longer treatment durations, strain-specific effects, or combinations with complementary therapeutic strategies.

Several factors should be considered when interpreting these findings. The high proportion of “unclear” ratings on the SYRCLE risk-of-bias tool likely reflects inherent reporting limitations commonly observed in animal studies rather than intrinsically poor methodological quality [77]. This pattern may be attributed to the structural characteristics of animal studies, where detailed reporting of procedures such as randomization and blinding is frequently omitted due to experimental constraints [78]. Accordingly, the biased results should be interpreted cautiously. Regarding publication bias, the funnel plots for TC and LDL-C suggest potential effect size overestimation. Although a trim-and-fill analysis was conducted to account for missing studies and adjust for this bias (Supplementary Table S3) [79], the LDL-C level reduction remained significant even after adjustment, indicating a robust LDL-C-lowering effect, even with a potential bias. In contrast, the significance of TC was attenuated following adjustment, suggesting that the observed TC-lowering effect should be interpreted cautiously, as it may be partially influenced by small-study effects. Nonetheless, overall heterogeneity across studies was relatively low, indicating consistent effects despite variations in animal models, dosages, and intervention durations. The use of single-strain interventions allowed assessment of individual strain-specific effects but precluded potentially synergistic interactions among multi-strain formulations. A major limitation of this meta-analysis is the paucity of data on *L. casei*. The inclusion of only one eligible study substantially limits the ability to draw definitive conclusions regarding its efficacy relative to *L. rhamnosus*. Translation to humans should be interpreted with caution given the use of static lipid endpoints and species differences in lipoprotein metabolism. Consequently, the current findings primarily support the effectiveness of *L. rhamnosus*, whereas conclusions regarding *L. casei* remain preliminary and require further investigation. In addition, confirmation of these findings in human studies is warranted. Despite these limitations, this meta-analysis provides a consolidated overview supporting the positive lipid-modulating effects of specific probiotic strains. Although this meta-analysis focused on supplementation with live bacteria, the implicated mechanisms, such as BSH activity and SCFAs production, indicate that the observed positive effects may be mediated by bioactive metabolites or structural components [80,81]. Heat-killed cells, categorized as postbiotics, have been increasingly recognized for their potential to retain lipid-lowering activity through residual enzymatic or structural functions [82]. Accordingly, future research should validate the clinical efficacy of live *L. rhamnosus* and explore whether postbiotic formulations can confer comparable benefits, potentially providing a more stable and practical approach to dyslipidemia management.

## 5. Conclusions

This systematic review and meta-analysis establish *L. rhamnosus* as an effective probiotic strain for lowering lipid levels, supporting its role as an adjunctive nutritional intervention for dyslipidemia management. *L. rhamnosus,* with limited evidence for *L. casei,* was associated with significant reductions in TG, TC, and LDL-C, indicating meaningful improvements in the atherogenic lipid profile. The heterogeneity observed across these outcomes (I^2^ = 36–51%) suggests consistent lipid-lowering effects across diverse experimental conditions. Collectively, these findings support the potential efficacy of *L. rhamnosus* in lipid regulation and reinforce the biological plausibility of probiotic-based interventions for dyslipidemia treatment. However, the evidence of publication bias observed for TC and LDL-C outcomes warrants cautious interpretation, as true effect sizes may differ from pooled estimates. In addition, the limited number of qualifying studies on *L. casei* precludes definitive conclusions regarding its relative effectiveness. Future research should prioritize rigorous investigations of *L. casei* to enable balanced and comparative analyses between probiotic strains.

Mechanistically, the lipid-lowering effects are likely attributable to gut microbiota-mediated modulation, such as bile acid deconjugation, SCFA production, and regulation of hepatic lipogenesis. These processes are mediated through the gut–liver axis, linking intestinal microbial activity to hepatic lipid metabolism. These mechanisms suggest that probiotic benefits may be limited to viable cells and mediated by bioactive metabolites and structural components. Collectively, these findings provide a scientific rationale for the development of targeted probiotic formulations and highlight postbiotics as alternative therapeutic strategies for dyslipidemia management. Well-designed human clinical trials are required to validate these preclinical findings and inform the development of next-generation synbiotic and postbiotic interventions.

## Figures and Tables

**Table 1:** Characteristics of the 12 selected animal studies.

Study ID	Year	Animal	Sex (M ^2)^/F ^3)^)	Age (wk)	*n*	Strain	Duration (wk ^1)^)	Dose (as Reported)	Disease Induction	Outcomes (Lipid Profile)
Nocianitri et al. [39]	2017	Wistar rats	M	-	6	*L. rhamnosus* SKG34, *L. rhamnosus* FBB42, *L. rhamnosus* (SKG34 + FBB42)	4	0.5 mL of cell suspension (10^8^ cells/mL)	HFD ^5)^	Serum TG ^10)^, TC ^11)^, LDL-C ^12)^, HDL-C ^13)^
Balakumar et al. [40]	2018	B6 ^4)^	M	8–10	6	*L. rhamnosus* GG	24	1.5 × 10^9^ colonies/mouse/day	HFD	Serum TG, TC, LDL-C, HDL-C
Park et al. [22]	2018	B6TacN	M	3	7–8	*L. rhamnosus* GG, *L. rhamnosus* BFE5264	9	1 × 10^10^ CFU ^9)^	HC ^6)^	Serum TG, TC, LDL-C, HDL-C
FANG et al. [41]	2019	ApoE −/− mice	M	8	3	*L. rhamnosus GR-1* (L) *L. rhamnosus GR-1* (H)	12	L: 5 × 10^7^ CFU H: 5 × 10^8^ CFU	HFCD ^7)^	Serum TG, TC, LDL-C, HDL-C
Sharma et al. [42]	2020	SD rats	M	6	7	*L. casei* ATCC 393, *L. rhamnosus* ATCC53103	12	1 × 10^9^ CFU	HFD	Plasma TG, TC LDL-C, HDL-C
Sun et al. [43]	2020	B6	F	4	12	*L. rhamnosus* LRa05	8	1 × 10^9^ CFU	HFD	Serum TG, TC, LDL-C, HDL-C
Choi et al. [44]	2021	B6	M	4	6	*L. rhamnosus* MG4502	8	2 × 10^8^ CFU	HFD	Serum TG, TC, LDL-C, HDL-C
Lee et al. [45]	2021	B6	M	10	6	*L. rhamnosus* 86	12	1 × 10^10^ CFU	HFD	Serum TG, TC, HDL-C
Özbek et al. [46]	2021	SD rats	M	6–8	8	*L. rhamnosus* GG	12	1 × 10^9^ CFU	HFD	Serum TC
Arellano-García et al. [38]	2023	Wistar rats	M	8–9	8–9	*L. rhamnosus* GG	6	1 × 10^9^ CFU	HFHF ^8)^	Serum TG
Melo et al. [47]	2023	Wistar rats	M	8–9	8–9	*L. rhamnosus* GG	6	1 × 10^9^ CFU	HFHF	Serum TG, TC, HDL-C
Ho et al. [48]	2024	B6	M	4	8	*L. rhamnosus* SG069	12	5 × 10^8^ CFU	HFD	Serum TG, TC, LDL-C

^1)^ wk: weeks; ^2)^ M: male, ^3)^ F: female; ^4)^ B6: C57BL/6 mice; ^5)^ HFD: high-fat diet; ^6)^ HC: high-cholesterol diet; ^7)^ HFCD: high-fat high-cholesterol diet; ^8)^ HFHF: high-fat high-fructose diet; ^9)^ CFU: colony forming unit; ^10)^ TG: triglyceride; ^11)^ TC: total cholesterol; ^12)^ LDL-C: low-density lipoprotein cholesterol; ^13)^ HDL-C: high-density lipoprotein cholesterol.

**Table 2:** Subgroup-specific pooled SMDs (95% CI), heterogeneity (I^2^), and tests for subgroup differences by intervention duration, species, and diet type.

Outcome	Moderator	Subgroup	k (Comparisons)	Pooled SMDs (95% CI ^1)^)	I^2^ (%)	*p*-Value (Subgroup Difference)
TG ^3)^	Duration	≥8 wk	10	−1.21 (−1.87 to −0.55)	57.1	0.3134
		<8 wk	5	−1.78 (−2.68 to −0.89)	32.8	
	Species	Mice	8	−1.02 (−1.75 to −0.29)	60.7	0.0985
		Rats	7	−1.86 (−2.53 to −1.19)	6.1	
	Diet type	HFD ^2)^	10	−1.56 (−2.14 to −0.97)	33.7	*p* < 0.05
		HFD + Fructose	2	−2.27 (−4.52 to −0.02)	81.2	
		Other/Combined	3	−0.22 (−1.02 to 0.59)	0.0	
TC ^4)^	Duration	≥8 wk	11	−0.87 (−1.44 to −0.31)	51.9	0.8303
		<8 wk	4	−0.78 (−1.48 to −0.07)	0.0	
	Species	Mice	8	−1.02 (−1.55 to −0.49)	34.5	0.3636
		Rats	7	−0.62 (−1.30 to 0.05)	35.8	
	Diet type	HFD	11	−1.01 (−1.59 to −0.42)	50.2	0.3027
		Other/Combined	4	−0.55 (−1.19 to 0.08)	0.0	
LDL-C ^5)^	Duration	≥8 wk	9	−1.39 (−1.98 to −0.81)	35.8	0.0503
		<8 wk	3	−2.99 (−4.48 to −1.50)	0.0	
	Species	Mice	7	−1.24 (−1.89 to −0.59)	40.7	*p* < 0.05
		Rats	5	−2.48 (−3.42 to −1.53)	0.0	
	Diet type	HFD	9	−1.88 (−2.58 to −1.17)	41.2	0.0892
		Other/Combined	3	−0.91 (−1.77 to −0.04)	0.0	
HDL-C ^6)^	Duration	≥8 wk	9	0.19 (−0.24 to 0.62)	23.2	0.9994
		<8 wk	4	0.19 (−1.38 to 1.76)	77.8	
	Species	Mice	7	0.24 (−0.30 to 0.77)	32.9	0.8509
		Rats	6	0.12 (−0.91 to 1.15)	66.9	
	Diet type	HFD	9	0.34 (−0.19 to 0.87)	32.6	0.3877
		Other/Combined	4	−0.26 (−1.49 to 0.98)	71.9	

^1)^ CI: confidence interval; ^2)^ HFD: high-fat diet; ^3)^ TG: triglyceride; ^4)^ TC: total cholesterol; ^5)^ LDL-C: low-density lipoprotein cholesterol; ^6)^ HDL-C: high-density lipoprotein cholesterol.

## Data Availability

The original contributions presented in the study are included in the article/Supplementary Material, further inquiries can be directed to the corresponding author.

## Supplementary Materials

The following supporting information can be downloaded at https://www.mdpi.com/article/10.3390/foods15030465/s1. Table S1: Database search strategy for animal studies evaluating the effects of *Lactobacillus rhamnosus* and *Lactobacillus lactis* on dyslipidemia; Table S2: Meta-regression results for intervention duration and probiotic dose as potential moderators of lipid outcomes; Table S3: Trim-and-fill adjusted effect sizes for each outcome; Figure S1: Funnel plots evaluating publication bias for (A) triglycerides (TG), (B) total cholesterol (TC), (C) low-density lipoprotein (LDL-C), and (D) high-density lipoprotein (HDL-C); Figure S2: Leave-one-out sensitivity analysis for (A) triglyceride (TG) and (B) high-density lipoprotein (HDL-C), evaluating the influence of each individual study on the pooled effect estimate; Figure S3: Baujat plots for (A) triglyceride (TG) and (B) high-density lipoprotein (HDL-C), highlighting the top three studies contributing disproportionately to heterogeneity and pooled effect size; Figure S4: Subgroup analyses according to intervention duration (<8 weeks vs. ≥8 weeks) for (A) triglyceride (TG), (B) total cholesterol (TC), (C) low-density lipoprotein cholesterol (LDL-C), and (D) high-density lipoprotein cholesterol (HDL-C); Figure S5: Subgroup analyses according to animal species (mice vs. rats) for (A) triglyceride (TG), (B) total cholesterol (TC), (C) low-density lipoprotein cholesterol (LDL-C), and (D) high-density lipoprotein cholesterol (HDL-C); Figure S6: Subgroup analyses according to diet type (e.g., HFD, HFHC, and HFHF) for (A) triglyceride (TG), (B) total cholesterol (TC), (C) low-density lipoprotein cholesterol (LDL-C), and (D) high-density lipoprotein cholesterol (HDL-C).

## Abbreviations

The following abbreviations are used in this manuscript:

TG	Triglyceride
TC	Total cholesterol
LDL-C	Low-density lipoprotein cholesterol
SMD	Standardized mean difference
CI	Confidence interval
HDL-C	High-density lipoprotein cholesterol
BSH	Bile salt hydrolase
SCFA	Short-chain fatty acid
LAB	Lactic acid bacteria
PRISMA	Preferred Reporting Items for Systematic Reviews and Meta-Analyses
PROSPERO	International Prospective Register of Systematic Reviews
SD	Standard deviation
SE	Standard error
PI	Prediction interval
