# Supplementary material for: Improving Lipid Profiles Through Lactobacillus rhamnosus Supplementation in Dyslipidemic Animal Models: A Systematic Review and Meta-Analysis
Source: Foods. 2026 Jan 29;15(3):465. doi: 10.3390/foods15030465 (PMC12897425; doi:10.3390/foods15030465)
Supplement: Supplementary file 1 [file foods-15-00465-s001.zip › foods-4111013-supplementary.pdf]

Supplemenatay Table S1. Database search strategy for animal studies evaluating the effects of *Lactobacillus rhamnosus* and *Lactobacillus lactis* on dyslipidemia

Databases	Search strategy	Record (N[1)])
Pubmed	(((gastrointestinal microbiome[MeSH Terms]) OR (Lactobacillus rhamnosus[Text Word])) OR (Lactobacillus casei[Text Word])) AND (dyslipidemias[MeSH Terms])	432
Cochrane library	(gastrointestinal microbiome) OR (Lactobacillus rhamnosus) OR (Lactobacillus casei) AND (dyslipidemias)	44,867
EMBASE	('intestine flora'/exp OR 'lactobacillus rhamnosus'/exp OR 'lactobacillus casei'/exp) AND 'dyslipidemia'/exp	21,280
Web of science	gastrointestinal microbiome (All Fields) OR Lactobacillus rhamnosus (All Fields) OR Lactobacillus casei (All Fields) AND dyslipidemias (All Fields)	1,280

[1)]N: number

Supplementary Table S2. Meta-regression results for intervention duration and probiotic dose as potential moderators of lipid outcomes

Outcome	Moderators	β (slope)[2]	95% CI[1]	*p*- value	R^2 (%)
**TG[3]**	Duration	-0.030	-0.16 to 0.10	$p = 0.644$	0.0
	Dose (log10)	0.019	-0.14 to 0.18	$p = 0.819$	0.0
**TC[4]**	Duration	-0.010	-0.11 to 0.09	$p = 0.844$	0.0
	Dose (log10)	0.106	-0.06 to 0.28	$p = 0.219$	8.1
**LDL-C[5]**	Duration	0.026	-0.10 to 0.15	$p = 0.670$	0.0
	Dose (log10)	0.128	-0.10 to 0.35	$p = 0.260$	4.7
**HDL-C[6]**	Duration	0.064	-0.04 to 0.17	$p = 0.239$	13.6
	Dose (log10)	0.042	-0.15 to 0.24	*p = 0.674*	0.0

[1]CI: confidence interval; [2]β: regression coefficient; [3]TG: triglyceride; [4]TC: total cholesterol; [5]LDL-C: low-density lipoprotein cholesterol; [6]HDL-C: high-density lipoprotein cholesterol

Supplementary Table S3. Trim-and-fill adjusted effect sizes for each lipid outcomes

Outcome	K (comparisons)	Egger's test *p*-value	Missing studies (filled)	Observed SMD[1)] (95% CI[2)])	Adjusted SMD (95% CI)
**TG[3)]**	15	$p = 0.156$	5	-1.380 (-1.974 to -0.789)	-0.804 (-1.508 to -0.100)
**TC[4)]**	15	$p < 0.05$	4	-0.849 (-1.322 to -0.376)	-0.537 (-1.079 to 0.006)
**LDL-C[5)]**	12	$p < 0.05$	3	-1.590 (-2.210 to -0.970)	-1.361 (-1.993 to -0.730)
**HDL-C[6)]**	13	$p = 0.1725$	3	0.183 (-0.412 to 0.778)	-0.113 (-0.740 to 0.514)

[1)]SMD: standardized mean difference; [2)]CI: confidence interval; [3)]TG: triglyceride; [4)]TC: total cholesterol; [5)]LDL-C: low-density lipoprotein cholesterol; [6)]HDL-C: high-density lipoprotein cholesterol

Supplementary Figure S1. Funnel plots evaluating publication bias for (A) triglyceride (TG), (B) total cholesterol (TC), (C) LDL-cholesterol (LDL-C), and (D) HDL-cholesterol (HDL-C).

(A)

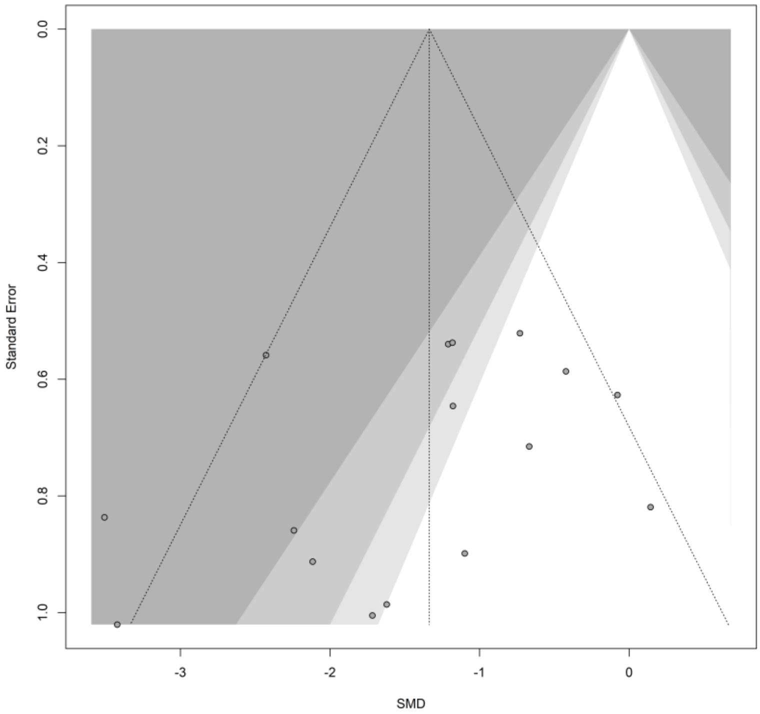

(B)

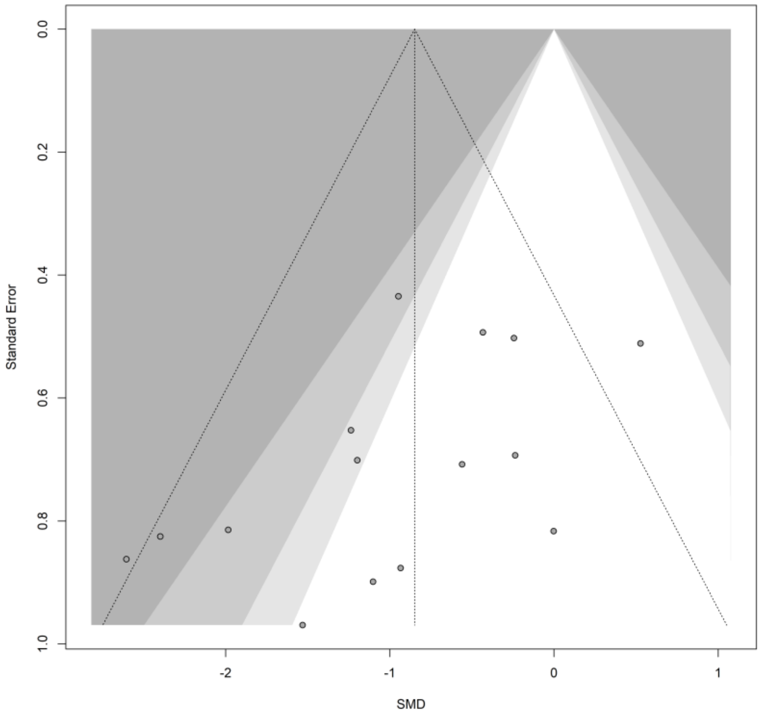

(C)

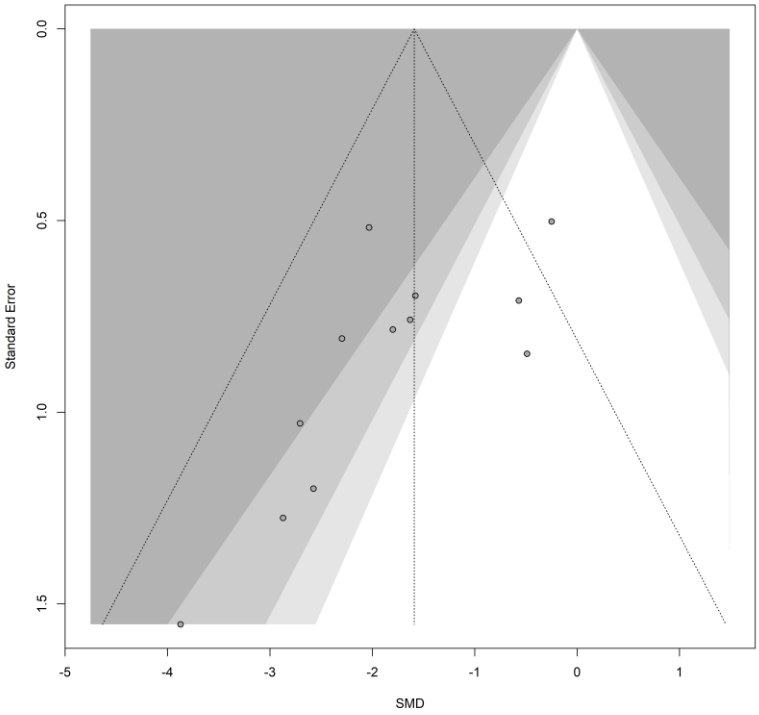

Standard Error
SMD
0.0
0.5
1.0
1.5
-5
-4
-3
-2
-1
0
1

(D)

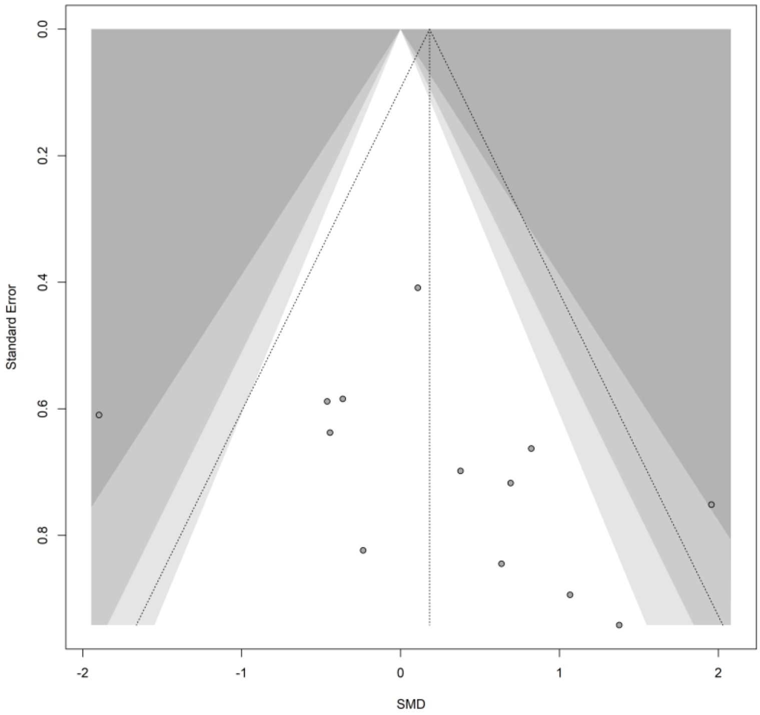

Standard Error
SMD
0.0
0.2
0.4
0.6
0.8
-2
-1
0
1
2

Supplementary Figure S2. Leave-one-out sensitivity analysis for (A) triglyceride (TG) and (B) high-density lipoprotein cholesterol (HDL-C), evaluating the influence of each individual study on the pooled effect estimate.

(A)

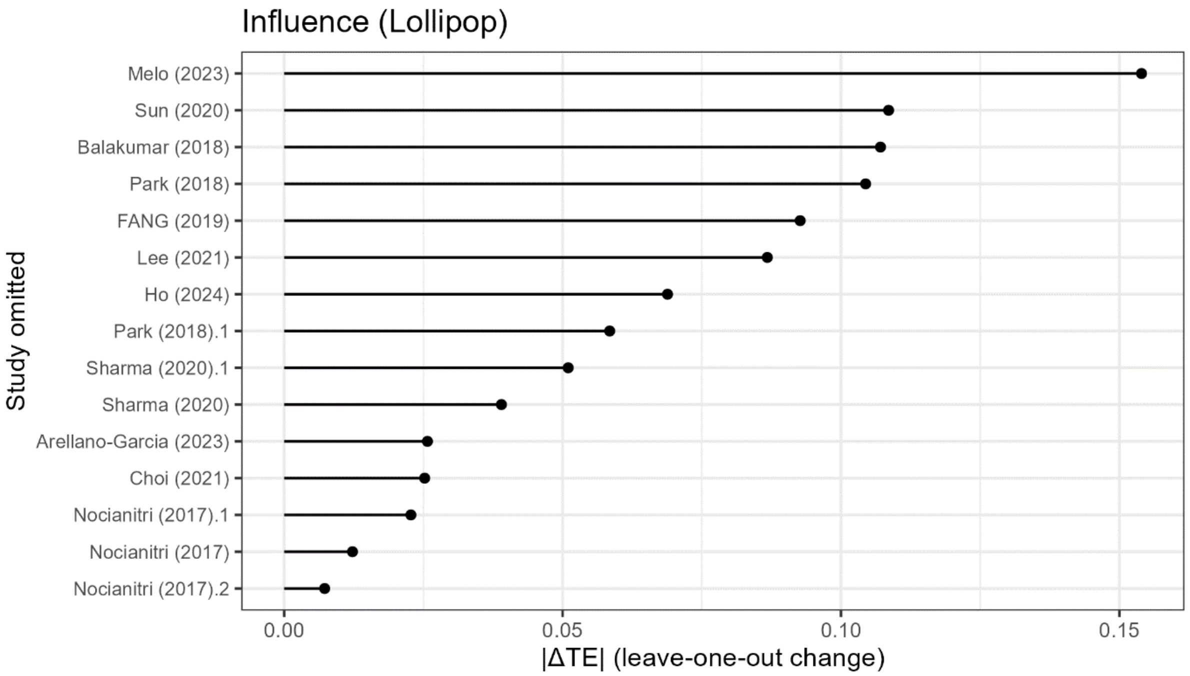

(B)

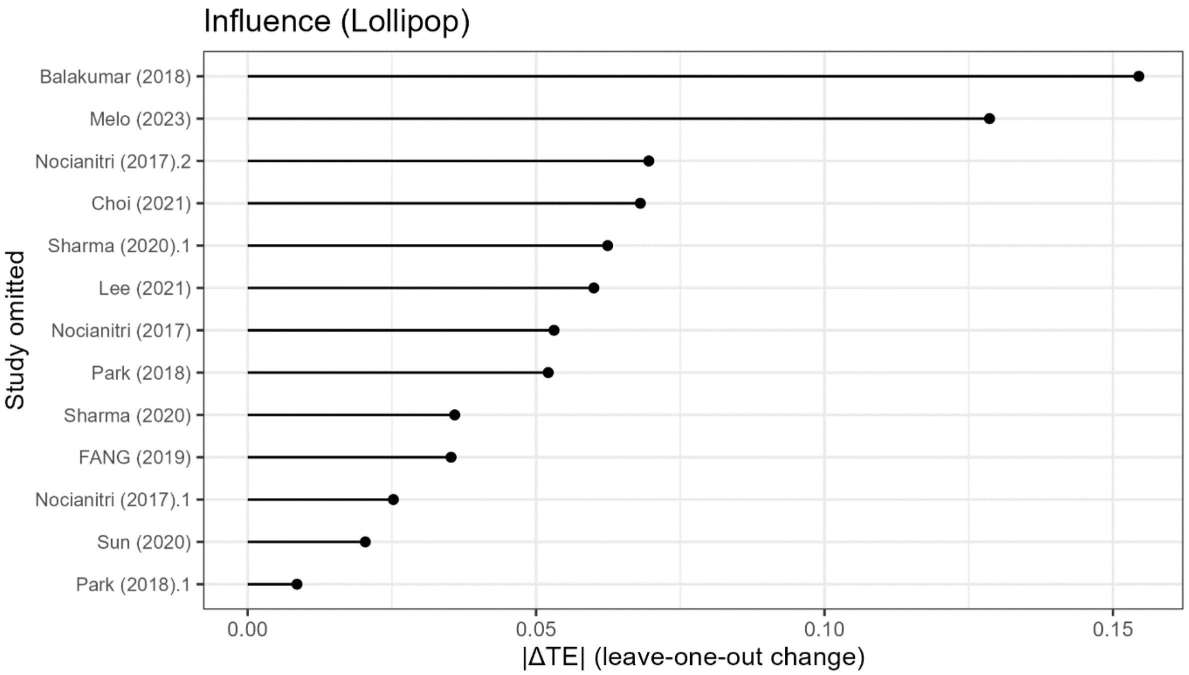

Supplementary Figure S3. Baujat plots for (A) triglyceride (TG) and (B) high-density lipoprotein cholesterol (HDL-C), highlighting the top three studies contributing disproportionately to heterogeneity and pooled effect size.

(A)

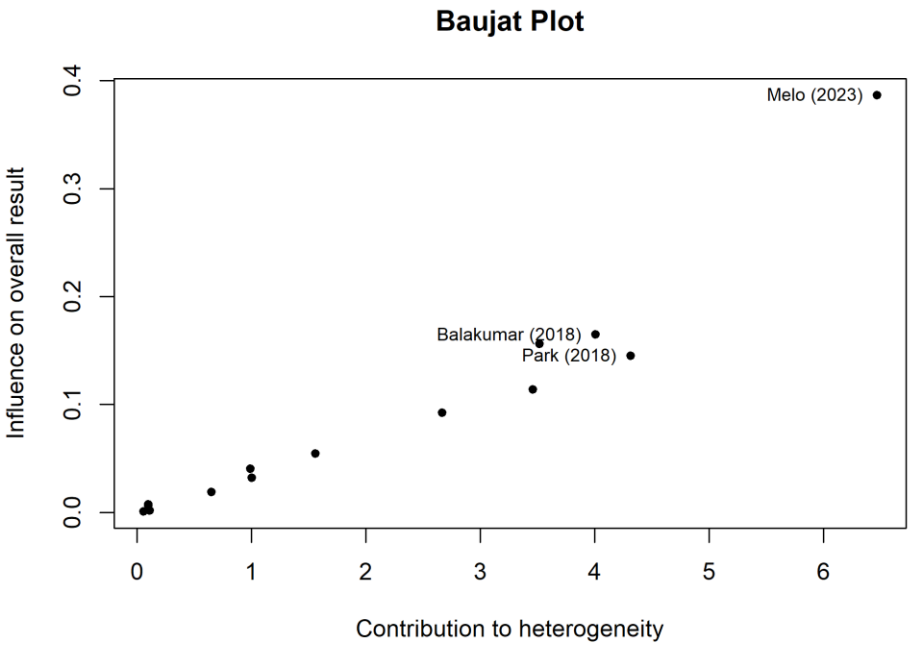

(B)

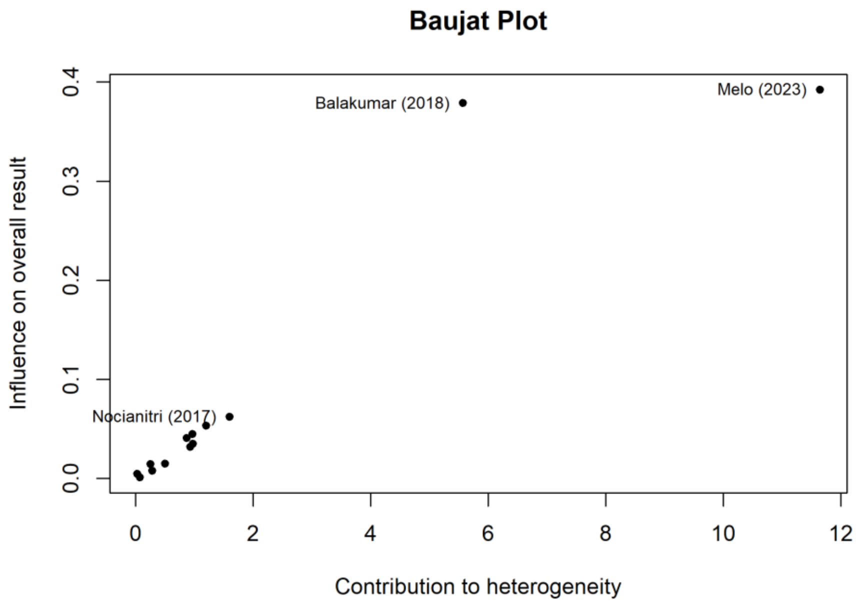

Supplementary Figure S4. Subgroup analyses according to intervention duration (< 8 weeks vs. ≥ 8 weeks) for (A) triglyceride (TG), (B) total cholesterol (TC), (C) low-density lipoprotein cholesterol (LDL-C), and (D) high-density lipoprotein cholesterol (HDL-C).

(A)

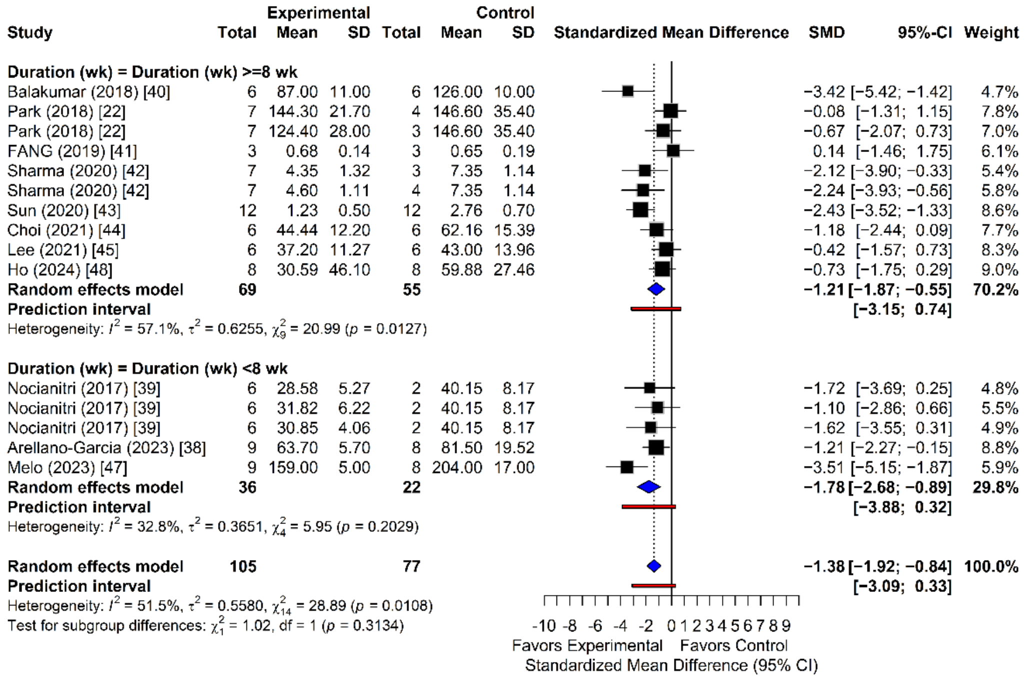

(B)

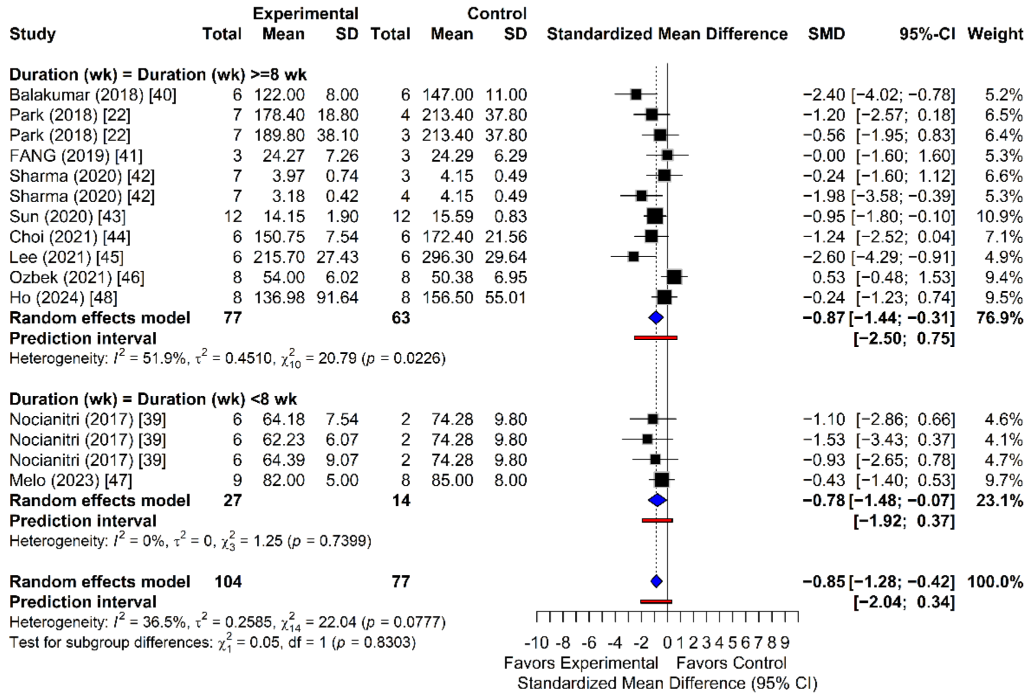

(C)

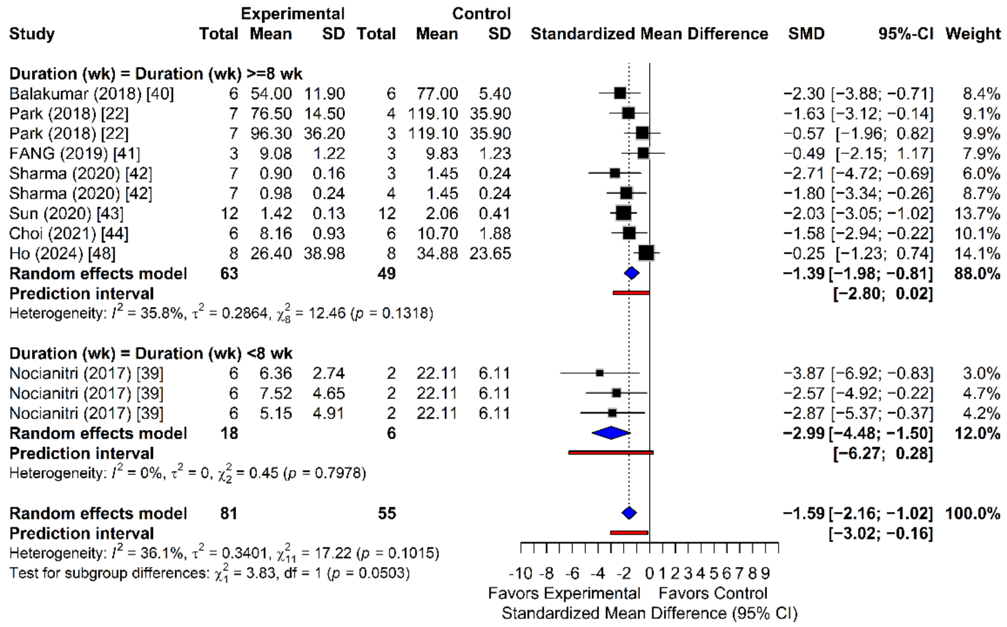
Study
Experimental
Total Mean SD
Control
Total Mean SD
Standardized Mean Difference
SMD 95%-CI Weight
Duration (wk) = Duration (wk) >=8 wk
Balakumar (2018) [40] 6 54.00 11.90 6 77.00 5.40 −2.30 [−3.88; −0.71] 8.4%
Park (2018) [22] 7 76.50 14.50 4 119.10 35.90 −1.63 [−3.12; −0.14] 9.1%
Park (2018) [22] 7 96.30 36.20 3 119.10 35.90 −0.57 [−1.96; 0.82] 9.9%
FANG (2019) [41] 3 9.08 1.22 3 9.83 1.23 −0.49 [−2.15; 1.17] 7.9%
Sharma (2020) [42] 7 0.90 0.16 3 1.45 0.24 −2.71 [−4.72; −0.69] 6.0%
Sharma (2020) [42] 7 0.98 0.24 4 1.45 0.24 −1.80 [−3.34; −0.26] 8.7%
Sun (2020) [43] 12 1.42 0.13 12 2.06 0.41 −2.03 [−3.05; −1.02] 13.7%
Choi (2021) [44] 6 8.16 0.93 6 10.70 1.88 −1.58 [−2.94; −0.22] 10.1%
Ho (2024) [48] 8 26.40 38.98 8 34.88 23.65 −0.25 [−1.23; 0.74] 14.1%
Random effects model 63 49 −1.39 [−1.98; −0.81] 88.0%
Prediction interval [−2.80; 0.02]
Heterogeneity: I^2 = 35.8%, τ^2 = 0.2864, χ^2_8 = 12.46 (p = 0.1318)
Duration (wk) = Duration (wk) <8 wk
Nocianitri (2017) [39] 6 6.36 2.74 2 22.11 6.11 −3.87 [−6.92; −0.83] 3.0%
Nocianitri (2017) [39] 6 7.52 4.65 2 22.11 6.11 −2.57 [−4.92; −0.22] 4.7%
Nocianitri (2017) [39] 6 5.15 4.91 2 22.11 6.11 −2.87 [−5.37; −0.37] 4.2%
Random effects model 18 6 −2.99 [−4.48; −1.50] 12.0%
Prediction interval [−6.27; 0.28]
Heterogeneity: I^2 = 0%, τ^2 = 0, χ^2_2 = 0.45 (p = 0.7978)
Random effects model 81 55 −1.59 [−2.16; −1.02] 100.0%
Prediction interval [−3.02; −0.16]
Heterogeneity: I^2 = 36.1%, τ^2 = 0.3401, χ^2_{11} = 17.22 (p = 0.1015)
Test for subgroup differences: χ^2_1 = 3.83, df = 1 (p = 0.0503)
-10 -8 -6 -4 -2 0 1 2 3 4 5 6 7 8 9
Favors Experimental Favors Control
Standardized Mean Difference (95% CI)

(D)

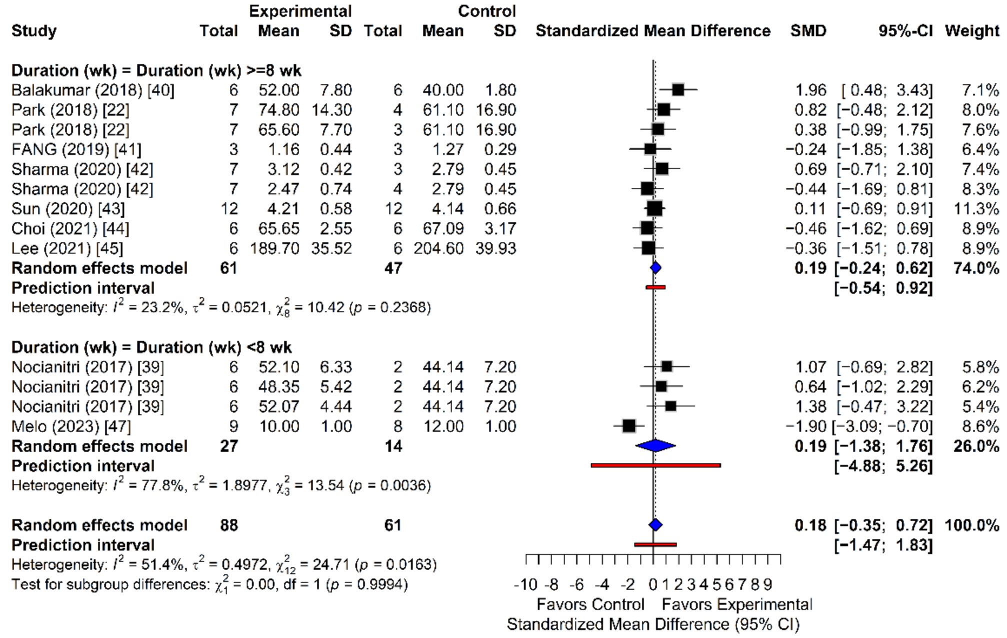
Study
Experimental
Total Mean SD
Control
Total Mean SD
Standardized Mean Difference
SMD 95%-CI Weight
Duration (wk) = Duration (wk) >=8 wk
Balakumar (2018) [40] 6 52.00 7.80 6 40.00 1.80 1.96 [0.48; 3.43] 7.1%
Park (2018) [22] 7 74.80 14.30 4 61.10 16.90 0.82 [−0.48; 2.12] 8.0%
Park (2018) [22] 7 65.60 7.70 3 61.10 16.90 0.38 [−0.99; 1.75] 7.6%
FANG (2019) [41] 3 1.16 0.44 3 1.27 0.29 −0.24 [−1.85; 1.38] 6.4%
Sharma (2020) [42] 7 3.12 0.42 3 2.79 0.45 0.69 [−0.71; 2.10] 7.4%
Sharma (2020) [42] 7 2.47 0.74 4 2.79 0.45 −0.44 [−1.69; 0.81] 8.3%
Sun (2020) [43] 12 4.21 0.58 12 4.14 0.66 0.11 [−0.69; 0.91] 11.3%
Choi (2021) [44] 6 65.65 2.55 6 67.09 3.17 −0.46 [−1.62; 0.69] 8.9%
Lee (2021) [45] 6 189.70 35.52 6 204.60 39.93 −0.36 [−1.51; 0.78] 8.9%
Random effects model 61 47 0.19 [−0.24; 0.62] 74.0%
Prediction interval [−0.54; 0.92]
Heterogeneity: I^2 = 23.2%, τ^2 = 0.0521, χ^2_8 = 10.42 (p = 0.2368)
Duration (wk) = Duration (wk) <8 wk
Nocianitri (2017) [39] 6 52.10 6.33 2 44.14 7.20 1.07 [−0.69; 2.82] 5.8%
Nocianitri (2017) [39] 6 48.35 5.42 2 44.14 7.20 0.64 [−1.02; 2.29] 6.2%
Nocianitri (2017) [39] 6 52.07 4.44 2 44.14 7.20 1.38 [−0.47; 3.22] 5.4%
Melo (2023) [47] 9 10.00 1.00 8 12.00 1.00 −1.90 [−3.09; −0.70] 8.6%
Random effects model 27 14 0.19 [−1.38; 1.76] 26.0%
Prediction interval [−4.88; 5.26]
Heterogeneity: I^2 = 77.8%, τ^2 = 1.8977, χ^2_3 = 13.54 (p = 0.0036)
Random effects model 88 61 0.18 [−0.35; 0.72] 100.0%
Prediction interval [−1.47; 1.83]
Heterogeneity: I^2 = 51.4%, τ^2 = 0.4972, χ^2_{12} = 24.71 (p = 0.0163)
Test for subgroup differences: χ^2_1 = 0.00, df = 1 (p = 0.9994)
-10 -8 -6 -4 -2 0 1 2 3 4 5 6 7 8 9
Favors Control Favors Experimental
Standardized Mean Difference (95% CI)

Supplementary Figure S5. Subgroup analyses according to animal species (mice vs. rats) for (A) triglyceride (TG), (B) total cholesterol (TC), (C) low-density lipoprotein cholesterol (LDL-C), and (D) high-density lipoprotein cholesterol (HDL-C).

(A)

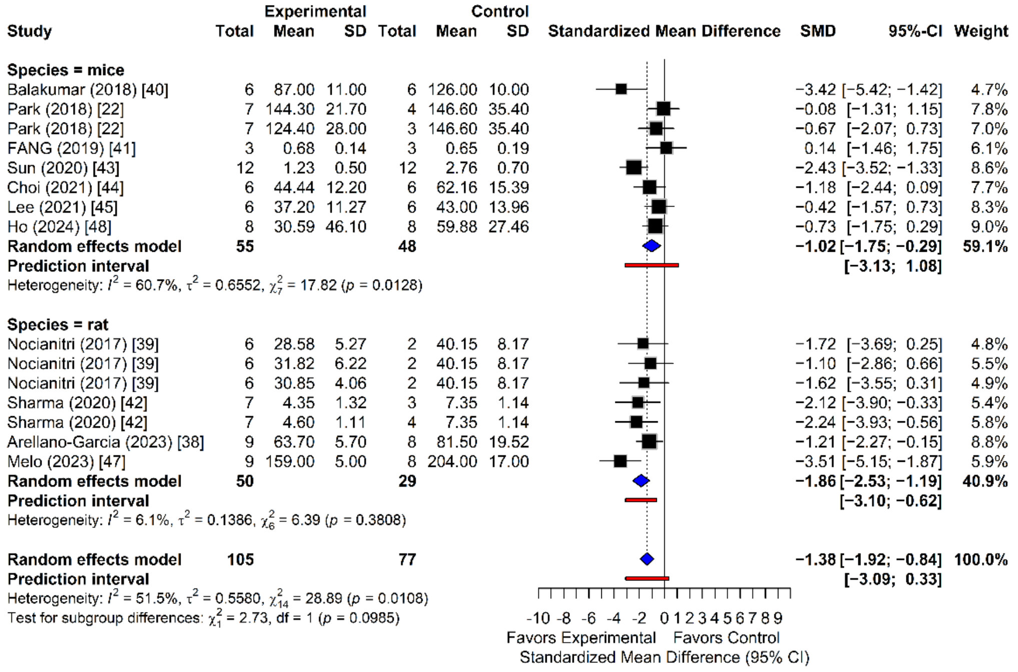

(B)

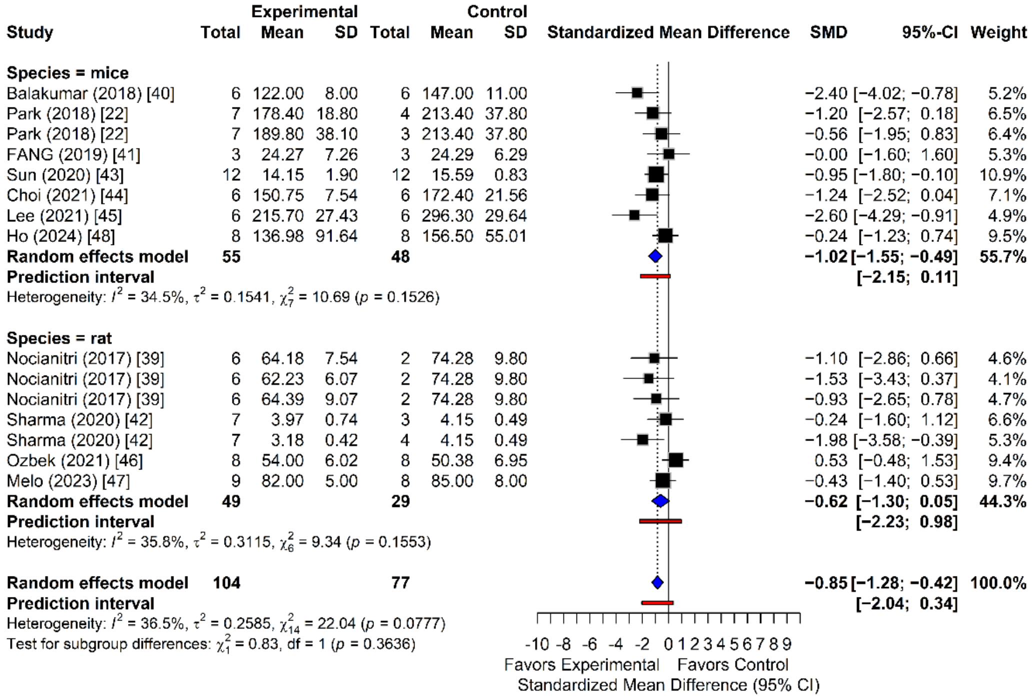

(C)

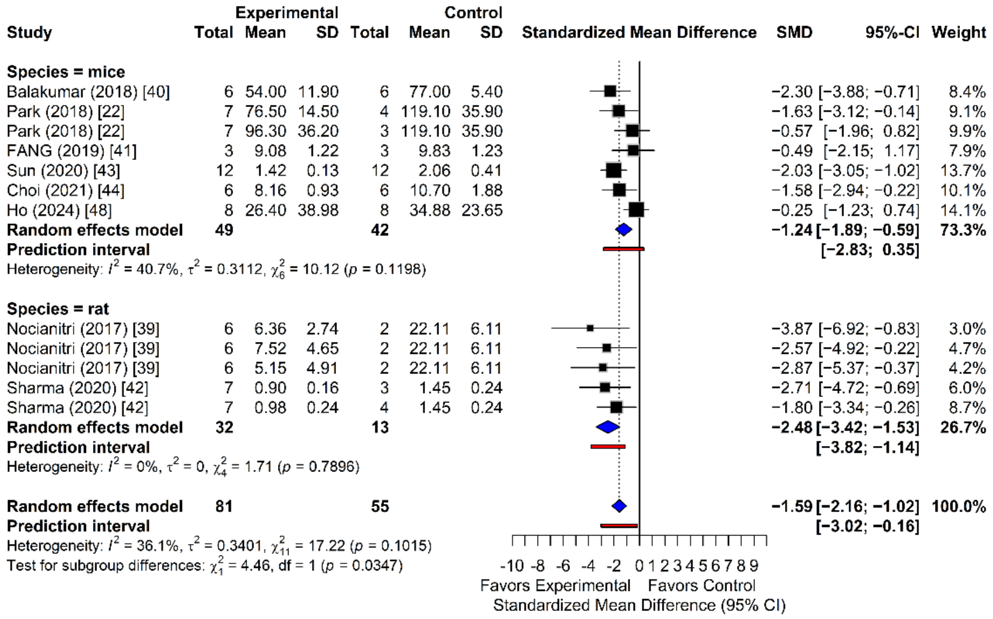
Experimental
Control
Study
Total Mean SD Total Mean SD
Standardized Mean Difference
SMD 95%-CI Weight
Species = mice
Balakumar (2018) [40] 6 54.00 11.90 6 77.00 5.40 −2.30 [−3.88; −0.71] 8.4%
Park (2018) [22] 7 76.50 14.50 4 119.10 35.90 −1.63 [−3.12; −0.14] 9.1%
Park (2018) [22] 7 96.30 36.20 3 119.10 35.90 −0.57 [−1.96; 0.82] 9.9%
FANG (2019) [41] 3 9.08 1.22 3 9.83 1.23 −0.49 [−2.15; 1.17] 7.9%
Sun (2020) [43] 12 1.42 0.13 12 2.06 0.41 −2.03 [−3.05; −1.02] 13.7%
Choi (2021) [44] 6 8.16 0.93 6 10.70 1.88 −1.58 [−2.94; −0.22] 10.1%
Ho (2024) [48] 8 26.40 38.98 8 34.88 23.65 −0.25 [−1.23; 0.74] 14.1%
Random effects model 49 42 −1.24 [−1.89; −0.59] 73.3%
Prediction interval [−2.83; 0.35]
Heterogeneity: I^2 = 40.7%, τ^2 = 0.3112, χ^2_6 = 10.12 (p = 0.1198)
Species = rat
Nocianitri (2017) [39] 6 6.36 2.74 2 22.11 6.11 −3.87 [−6.92; −0.83] 3.0%
Nocianitri (2017) [39] 6 7.52 4.65 2 22.11 6.11 −2.57 [−4.92; −0.22] 4.7%
Nocianitri (2017) [39] 6 5.15 4.91 2 22.11 6.11 −2.87 [−5.37; −0.37] 4.2%
Sharma (2020) [42] 7 0.90 0.16 3 1.45 0.24 −2.71 [−4.72; −0.69] 6.0%
Sharma (2020) [42] 7 0.98 0.24 4 1.45 0.24 −1.80 [−3.34; −0.26] 8.7%
Random effects model 32 13 −2.48 [−3.42; −1.53] 26.7%
Prediction interval [−3.82; −1.14]
Heterogeneity: I^2 = 0%, τ^2 = 0, χ^2_4 = 1.71 (p = 0.7896)
Random effects model 81 55 −1.59 [−2.16; −1.02] 100.0%
Prediction interval [−3.02; −0.16]
Heterogeneity: I^2 = 36.1%, τ^2 = 0.3401, χ^2_{11} = 17.22 (p = 0.1015)
Test for subgroup differences: χ^2_1 = 4.46, df = 1 (p = 0.0347)
-10 -8 -6 -4 -2 0 1 2 3 4 5 6 7 8 9
Favors Experimental Favors Control
Standardized Mean Difference (95% CI)

(D)

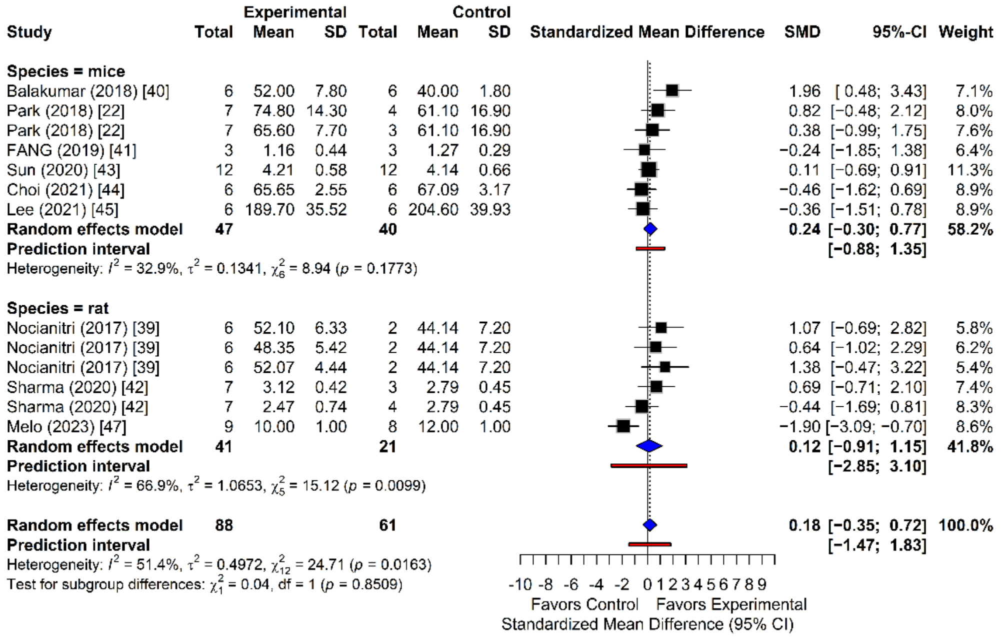
Experimental
Control
Study
Total Mean SD Total Mean SD
Standardized Mean Difference
SMD 95%-CI Weight
Species = mice
Balakumar (2018) [40] 6 52.00 7.80 6 40.00 1.80 1.96 [0.48; 3.43] 7.1%
Park (2018) [22] 7 74.80 14.30 4 61.10 16.90 0.82 [−0.48; 2.12] 8.0%
Park (2018) [22] 7 65.60 7.70 3 61.10 16.90 0.38 [−0.99; 1.75] 7.6%
FANG (2019) [41] 3 1.16 0.44 3 1.27 0.29 −0.24 [−1.85; 1.38] 6.4%
Sun (2020) [43] 12 4.21 0.58 12 4.14 0.66 0.11 [−0.69; 0.91] 11.3%
Choi (2021) [44] 6 65.65 2.55 6 67.09 3.17 −0.46 [−1.62; 0.69] 8.9%
Lee (2021) [45] 6 189.70 35.52 6 204.60 39.93 −0.36 [−1.51; 0.78] 8.9%
Random effects model 47 40 0.24 [−0.30; 0.77] 58.2%
Prediction interval [−0.88; 1.35]
Heterogeneity: I^2 = 32.9%, τ^2 = 0.1341, χ^2_6 = 8.94 (p = 0.1773)
Species = rat
Nocianitri (2017) [39] 6 52.10 6.33 2 44.14 7.20 1.07 [−0.69; 2.82] 5.8%
Nocianitri (2017) [39] 6 48.35 5.42 2 44.14 7.20 0.64 [−1.02; 2.29] 6.2%
Nocianitri (2017) [39] 6 52.07 4.44 2 44.14 7.20 1.38 [−0.47; 3.22] 5.4%
Sharma (2020) [42] 7 3.12 0.42 3 2.79 0.45 0.69 [−0.71; 2.10] 7.4%
Sharma (2020) [42] 7 2.47 0.74 4 2.79 0.45 −0.44 [−1.69; 0.81] 8.3%
Melo (2023) [47] 9 10.00 1.00 8 12.00 1.00 −1.90 [−3.09; −0.70] 8.6%
Random effects model 41 21 0.12 [−0.91; 1.15] 41.8%
Prediction interval [−2.85; 3.10]
Heterogeneity: I^2 = 66.9%, τ^2 = 1.0653, χ^2_5 = 15.12 (p = 0.0099)
Random effects model 88 61 0.18 [−0.35; 0.72] 100.0%
Prediction interval [−1.47; 1.83]
Heterogeneity: I^2 = 51.4%, τ^2 = 0.4972, χ^2_{12} = 24.71 (p = 0.0163)
Test for subgroup differences: χ^2_1 = 0.04, df = 1 (p = 0.8509)
-10 -8 -6 -4 -2 0 1 2 3 4 5 6 7 8 9
Favors Control Favors Experimental
Standardized Mean Difference (95% CI)

Supplementary Figure S6. Subgroup analyses according to diet type (e.g., HFD, HFHC, and HFHF) for (A) triglyceride (TG), (B) total cholesterol (TC), (C) low-density lipoprotein cholesterol (LDL-C), and (D) high-density lipoprotein cholesterol (HDL-C).

(A)

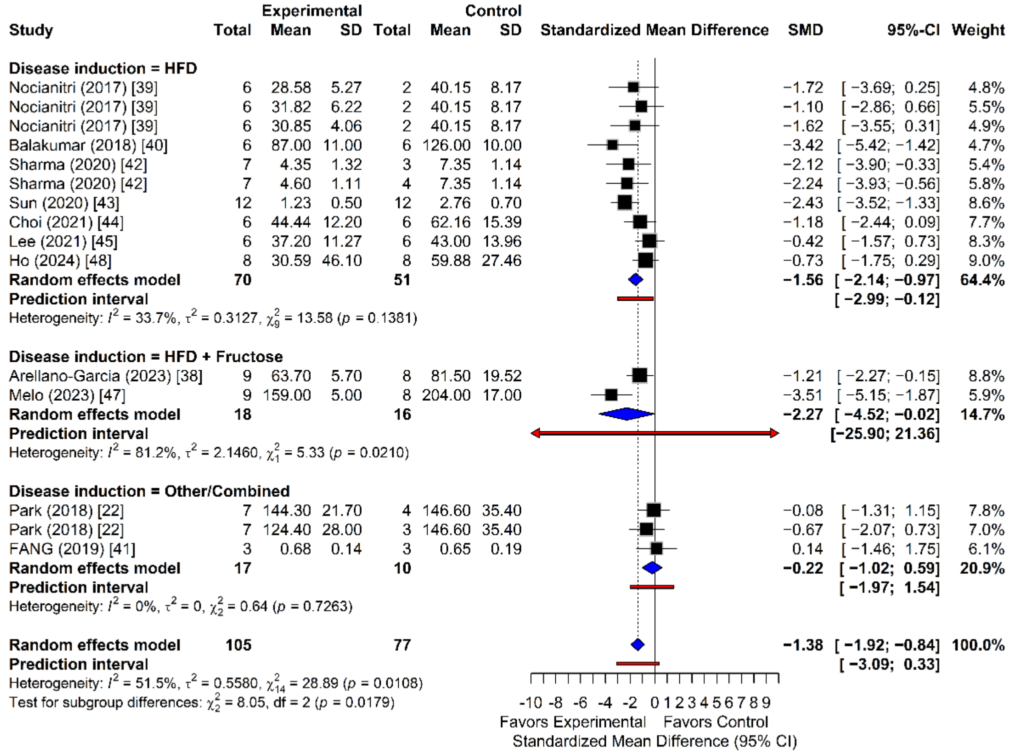

(B)

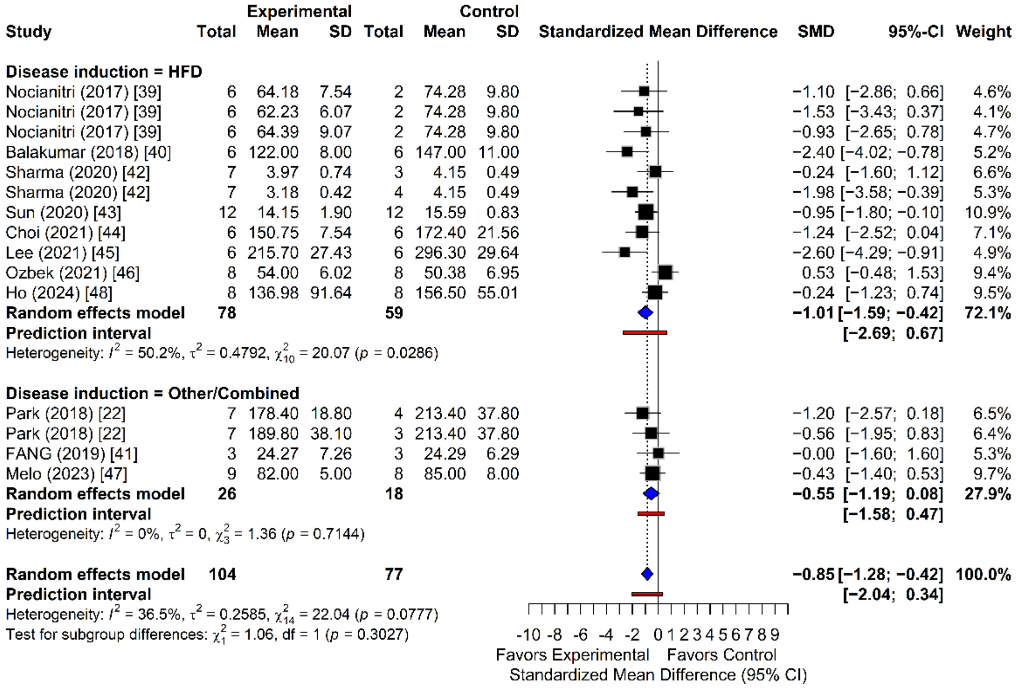

(C)

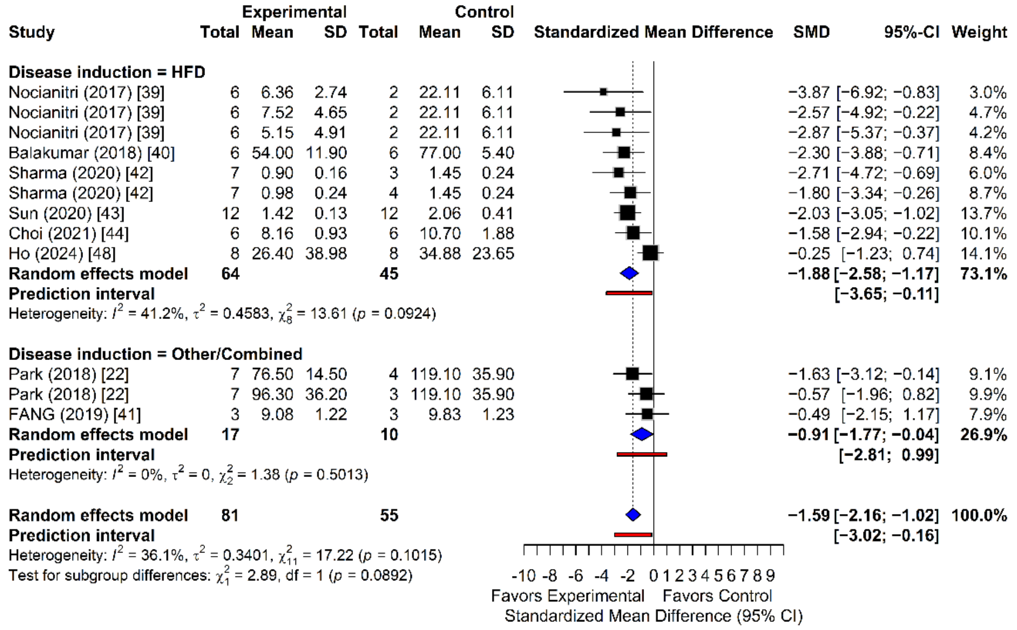

Experimental Control
Study Total Mean SD Total Mean SD Standardized Mean Difference SMD 95%-CI Weight
Disease induction = HFD
Nocianitri (2017) [39] 6 6.36 2.74 2 22.11 6.11 −3.87 [−6.92; −0.83] 3.0%
Nocianitri (2017) [39] 6 7.52 4.65 2 22.11 6.11 −2.57 [−4.92; −0.22] 4.7%
Nocianitri (2017) [39] 6 5.15 4.91 2 22.11 6.11 −2.87 [−5.37; −0.37] 4.2%
Balakumar (2018) [40] 6 54.00 11.90 6 77.00 5.40 −2.30 [−3.88; −0.71] 8.4%
Sharma (2020) [42] 7 0.90 0.16 3 1.45 0.24 −2.71 [−4.72; −0.69] 6.0%
Sharma (2020) [42] 7 0.98 0.24 4 1.45 0.24 −1.80 [−3.34; −0.26] 8.7%
Sun (2020) [43] 12 1.42 0.13 12 2.06 0.41 −2.03 [−3.05; −1.02] 13.7%
Choi (2021) [44] 6 8.16 0.93 6 10.70 1.88 −1.58 [−2.94; −0.22] 10.1%
Ho (2024) [48] 8 26.40 38.98 8 34.88 23.65 −0.25 [−1.23; 0.74] 14.1%
Random effects model 64 45 −1.88 [−2.58; −1.17] 73.1%
Prediction interval [−3.65; −0.11]
Heterogeneity: I^2 = 41.2%, τ^2 = 0.4583, χ^2_8 = 13.61 (p = 0.0924)
Disease induction = Other/Combined
Park (2018) [22] 7 76.50 14.50 4 119.10 35.90 −1.63 [−3.12; −0.14] 9.1%
Park (2018) [22] 7 96.30 36.20 3 119.10 35.90 −0.57 [−1.96; 0.82] 9.9%
FANG (2019) [41] 3 9.08 1.22 3 9.83 1.23 −0.49 [−2.15; 1.17] 7.9%
Random effects model 17 10 −0.91 [−1.77; −0.04] 26.9%
Prediction interval [−2.81; 0.99]
Heterogeneity: I^2 = 0%, τ^2 = 0, χ^2_2 = 1.38 (p = 0.5013)
Random effects model 81 55 −1.59 [−2.16; −1.02] 100.0%
Prediction interval [−3.02; −0.16]
Heterogeneity: I^2 = 36.1%, τ^2 = 0.3401, χ^2_{11} = 17.22 (p = 0.1015)
Test for subgroup differences: χ^2_1 = 2.89, df = 1 (p = 0.0892)
-10 -8 -6 -4 -2 0 1 2 3 4 5 6 7 8 9
Favors Experimental Favors Control
Standardized Mean Difference (95% CI)

(D)

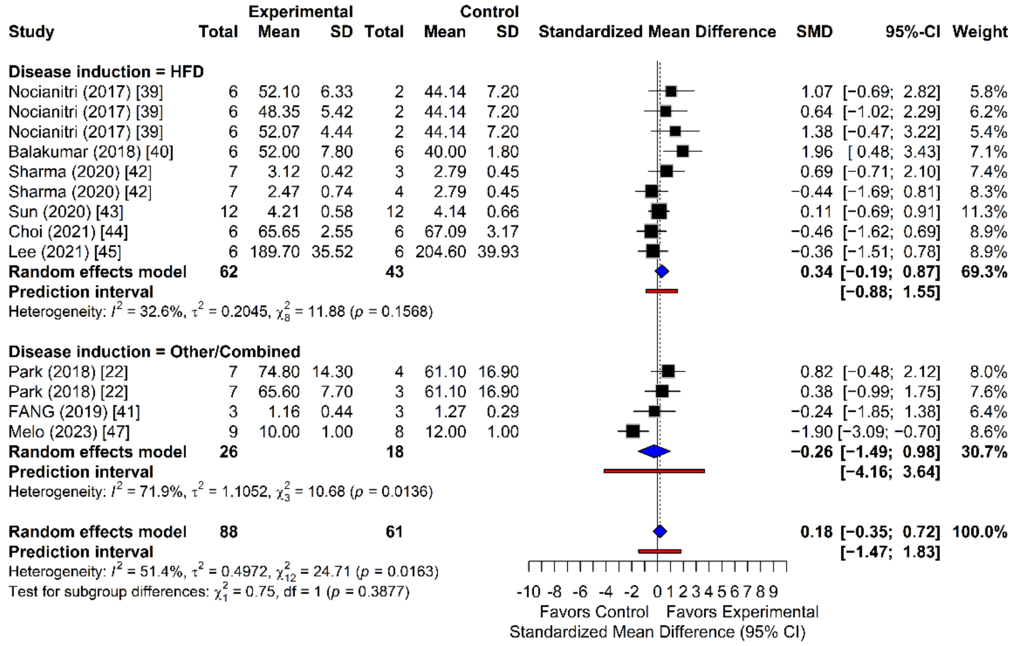

Experimental Control
Study Total Mean SD Total Mean SD Standardized Mean Difference SMD 95%-CI Weight
Disease induction = HFD
Nocianitri (2017) [39] 6 52.10 6.33 2 44.14 7.20 1.07 [−0.69; 2.82] 5.8%
Nocianitri (2017) [39] 6 48.35 5.42 2 44.14 7.20 0.64 [−1.02; 2.29] 6.2%
Nocianitri (2017) [39] 6 52.07 4.44 2 44.14 7.20 1.38 [−0.47; 3.22] 5.4%
Balakumar (2018) [40] 6 52.00 7.80 6 40.00 1.80 1.96 [0.48; 3.43] 7.1%
Sharma (2020) [42] 7 3.12 0.42 3 2.79 0.45 0.69 [−0.71; 2.10] 7.4%
Sharma (2020) [42] 7 2.47 0.74 4 2.79 0.45 −0.44 [−1.69; 0.81] 8.3%
Sun (2020) [43] 12 4.21 0.58 12 4.14 0.66 0.11 [−0.69; 0.91] 11.3%
Choi (2021) [44] 6 65.65 2.55 6 67.09 3.17 −0.46 [−1.62; 0.69] 8.9%
Lee (2021) [45] 6 189.70 35.52 6 204.60 39.93 −0.36 [−1.51; 0.78] 8.9%
Random effects model 62 43 0.34 [−0.19; 0.87] 69.3%
Prediction interval [−0.88; 1.55]
Heterogeneity: I^2 = 32.6%, τ^2 = 0.2045, χ^2_8 = 11.88 (p = 0.1568)
Disease induction = Other/Combined
Park (2018) [22] 7 74.80 14.30 4 61.10 16.90 0.82 [−0.48; 2.12] 8.0%
Park (2018) [22] 7 65.60 7.70 3 61.10 16.90 0.38 [−0.99; 1.75] 7.6%
FANG (2019) [41] 3 1.16 0.44 3 1.27 0.29 −0.24 [−1.85; 1.38] 6.4%
Melo (2023) [47] 9 10.00 1.00 8 12.00 1.00 −1.90 [−3.09; −0.70] 8.6%
Random effects model 26 18 −0.26 [−1.49; 0.98] 30.7%
Prediction interval [−4.16; 3.64]
Heterogeneity: I^2 = 71.9%, τ^2 = 1.1052, χ^2_3 = 10.68 (p = 0.0136)
Random effects model 88 61 0.18 [−0.35; 0.72] 100.0%
Prediction interval [−1.47; 1.83]
Heterogeneity: I^2 = 51.4%, τ^2 = 0.4972, χ^2_{12} = 24.71 (p = 0.0163)
Test for subgroup differences: χ^2_1 = 0.75, df = 1 (p = 0.3877)
-10 -8 -6 -4 -2 0 1 2 3 4 5 6 7 8 9
Favors Control Favors Experimental
Standardized Mean Difference (95% CI)
